# *Vitellogenin-3-like* and *Vitellogenin receptor* Genes Involved in the Regulation of Ovarian Development and Oviposition in *Diaphorina citri*

**DOI:** 10.3390/insects17060562

**Published:** 2026-05-29

**Authors:** Fang Wang, Xiaoyun Wang, Xialin Zheng, Zaixin Huang, Jinzi Wang, Xupo Ding, Hongcun Liu, Hailin Li

**Affiliations:** 1 Guangxi Key Laboratory of Polysaccharide Materials and Modifcation, School of Marine Sciences and Biotechnology, Guangxi Minzu University, Nanning 530008, China; m15677142501@163.com (F.W.); huangzx0526@163.com (Z.H.); wangjinzi@gxmzu.edu.cn (J.W.); xupoding@hotmail.com (X.D.); gxunliuhongcun@163.com (H.L.); 2 Guangxi Key Laboratory of Agrio-Environment and Agric-Product Safety, College of Agriculture, Guangxi University, Nanning 530004, China; wxy8771@163.com (X.W.); zheng-xia-lin@163.com (X.Z.)

**Keywords:** *D. citri*, *vitellogenin*, *vitellogenin receptor*, spatiotemporal expression, ovarian development, oviposition behavior

## Abstract

*Diaphorina citri* (Kuwayama) is one of the primary vectors of *Citrus Huanglongbing* (HLB), which poses a danger to the long-term development of the citrus industry. In this work, the spatiotemporal expression processes of the genes encoding *vitellogenin-3-like* (*Vg4*) and *vitellogenin receptor* (*VgR*) in *D. citri* females during the 1- to 30-day developmental stage of ovarian development and oviposition behavior were investigated. *Vg4* and *VgR* gene expression largely supports oocyte maturation in *D. citri* females at the 1- to 15-day developmental stage. During the 15- to 30-day developmental stage, the expression of the *Vg4* and *VgR* genes predominantly promotes the progression of oviposition behavior. Comparisons of ovarian development and oviposition behavior revealed that in the 15- to 30-day developmental stage, female egg production accounted for 75–85% of total oviposition, while mature oocyte formation occurred largely after the 15th day of ovarian development. The findings of this study provide fresh theoretical support and insights into the use of RNA interference (RNAi) technology to reduce the damage caused by *D. citri* populations.

## Simple Summary

*Diaphorina citri* (Kuwayama) female ovary development and oviposition behavior are dependent on yolk accumulation, which is defined by vitellogenin protein synthesis in the fat body, blood transport to the ovaries, and entry into the oocytes via vitellogenin receptor-mediated endocytosis to form yolk. We created expression profiles of ovarian *Vg* and *VgR* gene family members over the 1- to 30-day developmental stage and then utilized the IPS approach to apply RNAi interference to *Vg4* and *VgR* gene expression. The inhibiting *Vg4* and *VgR* gene expression inhibited ovarian development and oviposition behavior. Our findings suggest that the *Vg4* and *VgR* genes play critical roles in ovarian development and oviposition behavior.

## 1. Introduction

Vitellogenin (Vg) is an essential insect glycoprotein that controls ovarian development, oocyte production, and oviposition behavior [1,2]. The vitellogenin receptor (VgR) is a key carrier protein that controls the entry of Vg proteins into oocytes; VgR activities include transporting Vg and regulating ovarian development and signal transduction, with a specific focus on controlling ovarian maturation in female insects [3,4]. Therefore, understanding how the *vitellogenin* (*Vg*) and *vitellogenin receptor* (*VgR*) genes govern ovarian development and oviposition could provide a theoretical basis to develop molecular pesticides.

Insect *Vg* and *VgR* genes play important roles in ovarian development by mediating nutrient accumulation and maturation in oocytes as well as influencing the oviposition [5,6,7]. Previous studies illustrated that Vg proteins are generated in fat bodies and their synthesis is affected by hormones such as ecdysone and juvenile hormone (JH) [8,9]. Following synthesis, Vg proteins are transported via the bloodstream to the ovaries, where they accumulate outside the oocytes. After binding to the VgR receptor, expressed specifically on the oocyte membrane, Vg proteins enter the oocyte interior via VgR-mediated endocytosis to form the yolk sac that provides nutrients for embryonic development [10]. These results indicate that the coexpression of *Vg* and *VgR* genes is critical for ovarian development and suggest that RNA interference (RNAi) targeting either the *Vg* or *VgR* gene could result in abnormal ovarian development, oocyte degeneration, or a significant reduction in egg production [11,12,13]. For instance, interfering with the expression of the *Vg* and *VgR* genes in *Lasioderma serricorne* (Fabricius) resulted in considerably shorter average female oocyte lengths (203.7 µm; 228.9 µm; 226.5 µm) than those in the control check (CK) (416.5 µm) [14]. Although previous research has revealed partial functions of *Vg* and *VgR* genes using microinjection and RNAi technologies, the processes through which these genes influence ovarian development and oviposition behavior are long-term [15,16,17], especially for insect species with long longevity. Taking *Helicoverpa armigera*, *Apis mellifera*, *Leptopilina boulardi*, and *Bactrocera dorsalis* as an example, short-term RNAi of these insects *Vg* and *VgR* genes via microinjection may be insufficient to fully understand the intricate activities of these genes in regulating ovarian development and oviposition [18,19,20,21]. Given the complexity of temporal and spatial expression of the *Vg* and *VgR* gene family, more studies focused on the regulatory mechanisms that govern ovarian development and oviposition are needed.

*Diaphorina citri* (Kuwayama) is one of the primary vectors of *Citrus Huanglongbing* (HLB) [22,23]. The rapid spread of HLB in the world is due primarily to the high reproductive capacity and rapid spread of *D. citri* causing extremely serious economic losses worldwide annually [24,25,26,27,28,29,30,31]. Currently, one of the effective measures to minimize the impact of HLB has been based on area-wide suppression of *D. citri* population reproduction and dispersal [32]. However, *D. citri* population has a high reproductive capacity and significant overlap between generations, and, due to the heavy use of pesticides, *D. citri* has strong pesticide resistance, meaning that long-term management of this pest has yet to be established [33,34,35,36]. For example, partial *D. citri* females can live for 40 days [37], resulting in a higher egg production (312.50 ± 4.92 eggs per female) to increase the extent of its devastation [38]. Therefore, suppression of oviposition may be an effective technology to manage the *D. citri* population. Our preliminary research conducted high-throughput transcriptome sequencing on the abdomens of *D. citri* female, obtaining the *vitellogenin-1-like-1* (*Vg1*), *vitellogenin-1-like-2* (*Vg2*), *vitellogenin-2-like* (*Vg3*), *vitellogenin-3-like* (*Vg4*), *vitellogenin-like* (*Vg5*), and *vitellogenin receptor* (*VgR*) genes in the abdomens of females [39]. We further discovered that the Vg4 protein, corresponding to the *Vg4* gene, had the most complex amino acid structure in the *Vg* gene family and was highly expressed in the female abdomen [39]. This finding suggests that the *Vg4* gene may play a crucial role in egg formation physiology. RNA interference (RNAi) research revealed that the IPS (in-plant-system) technique for the delivery of double-stranded *vitellogenin-3-like* (*dsVg4*) and double-stranded *vitellogenin receptor* (*dsVgR*) strongly inhibited oviposition in *D. citri* females, and female *Vg4* and *VgR* genes were interfered with, resulting in incomplete eggs and nymphal abnormalities [13,40], which indicates that the *Vg* and *VgR* gene families are excellent candidates for critical target genes to decrease female oviposition. However, the *D. citri Vg4* and *VgR* genes regulating ovarian development and oviposition mechanisms remain unclear.

In this study, we used qRT–PCR to investigate the spatiotemporal expression patterns of *Vg* and *VgR* gene family members in *D. citri* female ovaries during the 1- to 30-day developmental stage. Furthermore, we used RNAi technology to investigate the functions of *D. citri Vg4* and *VgR* genes in ovarian development and oviposition. Our findings could provide a solid theoretical foundation to understand how the *Vg* and *VgR* genes influence ovarian development and oviposition and to develop novel techniques to achieve the sustainable management of *D. citri*.

## 2. Materials and Methods

### 2.1. Rearing and Collection

The population of *D. citri* was collected on a planting base (*Murraya exotica* L.) in Guangxi Minzu University (Guangxi Zhuang Autonomous Region, China). Adults were fed with *M. exotica* and stably passaged for more than three generations in an artificial climate chamber at 25 ± 1 °C with 75 ± 5% relative humidity and a light/dark (L:D) cycle = 14:10 h. Male and female adults emerging on the same day were randomly selected for the following experiments. For the RNAi assay, dsRNA was dissolved in water, added to the tube, and then transported to the tender shoot through the xylem of *M. exotica* for feeding the females. Oviposition among the females in the rearing container was observed and recorded.

Females in different treatment groups were reared in plastic containers. For the fabrication and usage of plastic containers, refer to [13]. For the RNAi assay, dsRNA was dissolved in water, added to the tube, and then transported to the tender shoot through the xylem of *M. exotica* for feeding the females. Oviposition of adults in the rearing container were observed and recorded.

### 2.2. DsRNA Synthesis

Ovaries of *D. citri* were dissected as described above and immediately frozen in liquid nitrogen in a 1.5 mL Eppendorf tube for total RNA extraction using TransZol Up (TransGen Biotech, Beijing, China). Afterward, cDNA synthesis was carried out using the PrimescriptTM RT reagent kit with gDNA eraser (perfect real time) (TaKaRa, Shiga, Japan) following the manufacturer’s instructions: (1) prepare the following mixture in an RNase-free EP tube: RNase-free ddH_2_O (up to 16 µL), 4 µL of 4 × 2-Step Gdna Erase-Out Mix, total RNA (1 pg–1 µg), 42 °C reaction for 2 min; (2) add 4 µL of 5×ToloScript qRT EasyMix directly to the reaction solution in step 1, and conduct reverse transcription, 37 °C for 15 min, 85 °C for 5 s. *Vg4* and *VgR* gene interference fragments were designed using SnapDragon-dsRNA Design https://www.flyrnai.org (accessed on 25 June 2022) (DRSC/TRiP Functional Genomics Resources & DRSC-BTRR, Harvard Medical School). Using the nucleotide sequences of the *Vg4* and *VgR* genes, primers were designed for PCR, quantitative real-time PCR (qRT–PCR) and synthetic dsRNA primers from the National Center for Biotechnology Information (NCBI, Bethesda, MD, USA) (Table 1). *Vg4* (477 bp) cDNA fragments were generated with the primer pair *Vg4* F3/R3 using 2×FastPfu Premix (TOLOBIO, Central lslip, NY, USA). The purified PCR-generated *Vg4* fragments were subsequently cloned and inserted into the pMD18T vector (DH5α; Thermo Fisher Scientific, Waltham, MA, USA). The resulting *Vg4* plasmids were used as templates to generate *dsVg4* with a dsRNA synthesis kit (T7 RiboMAX^TM^ Express RNAi System, Beijing, China; Supplementary Materials). The synthesis method is described in the manufacturer’s instructions: preparation of dsRNA synthesis (RiboMAX^TM^ Express T7 2X Buffer*, 10.0 μL; transcriptional template, 6.0 μL; DEPC water, 2.0 μL; Enzyme Mix T7 Express, 2.0 μL), in vitro transcription, reaction at 37 °C for 30 min; after holding at 70 °C for 10 min, hold at room temperature for 20 min; after standing at 37 °C for 30 min, place on ice for 5 min; and centrifuge for 10 min and anneal to obtain dsRNA. The synthesized dsRNA was quantified using an N80 Touch nanophotometer (Implen, München, Germany) at 260 nm, and the integrity was analyzed by agarose gel electrophoresis. The syntheses of *dsVgR* (346 bp) and *dsGFP* (726 bp) were carried out as described for *dsVg4*; the fragments amplified by the qRT-PCR primers used for the *Vg4* and *VgR* genes are located outside the RNAi target sequences, making them well-suited for evaluating the effectiveness of RNAi interference. The gene qRT-PCR amplification fragments can be found in the Supplementary Materials.

### 2.3. Spatiotemporal Expression

Seven rearing containers were prepared, with each rearing container holding 15 pairs of one-day-old females and males; the specific specifications and characteristics of the rearing containers have been described by [13]. All females from one container were selected every 5 days at 8 am and dissected for ovary sampling, while *M. exotica* tender shoots (Height 8 cm) and sterile water (500 μL) were replaced in other containers. The 15 female-dissected ovaries were first photographed with a microscopic measurement system coupled to a stereoscope (SMZ800N, Nikon, Tokyo, Japan) for further mature oocyte measurements, and then we merged 15 female-dissected ovaries to be sampled for RNA extraction. For the anatomical and measurement methods for the ovary, refer to [13]: measure 10 oocytes from the per-female ovaries, and record the mature oocyte number, as well as oocyte length and width (µm). In this way, ovaries were collected from 1-, 5-, 10-, 15-, 20-, 25-, and 30-day-old females. The experimental procedure was repeated three times. A total of 315 pairs of adult males and females are required. The cDNA from these samples was prepared as previously described and used for *Vg1*, *Vg2*, *Vg3*, *Vg4*, *Vg5*, *VgR* gene qRT–PCR assays (Supplementary Materials). qRT–PCR was carried out using 2 × Q3 SYBR qPCR Master Mix (High Rox) (TOLOBIO, Shanghai, China) following the manufacturer’s instructions: (1) prepare the following mixture in an RNase-free Eppendorf tube: 10 μL of 2 × Q3 SYBR qPCR Master Mix, 0.4 μL of primer 1 (10 µM), 0.4 μL of primer 2 (10 µM), 1 μL of template cDNA, and 8.2 μL of ddH_2_O; (2) place the prepared solution into 96-well plates and perform qRT–PCR on a qRT–PCR machine. The qRT–PCR conditions were as follows: predenaturation, cycle 1 at 95 °C for 30 s; circular reaction, cycle 40 at 95 °C for 10 s; melting curves, cycle 1 at 95 °C for 15 s, 60 °C for 60 s, and 95 °C for 15 s. All reactions were performed with the QuantStudio^TM^ Real-Time PCR system (Applied Biosystems, Waltham, MA, USA) using the primers listed in Table 1. Relative gene expression data of all treatment groups from ovaries qRT–PCR were analyzed using the dual internal reference gene *Actin* and *Elongation Factor 1-alpha* (*EF1a*) 2^−ΔΔCT^ method. QRT-PCR was performed for each gene with three biological replicates and three technical replicates (Supplementary Materials).

### 2.4. RNAi for Ovarian Development

Six rearing containers were prepared, with each rearing container holding 15 pairs of one-day-old females and males. *M. exotica* tender shoots and 300 μL *dsVg4* solution (1 ng/μL) were placed in containers, while 15 females from one container were selected every 5 days and dissected for ovary sampling, and the solution and *M. exotica* tender shoots were replaced. For the anatomical and measurement methods for the ovary, refer to [13]. Refer to the method for the experiment for the spatiotemporal expression of *Vg* and *VgR* genes for photographing and measuring the ovaries. Then, 15 female ovaries were sampled for RNA extraction. In this way, ovaries were collected from 5-, 10-, 15-, 20-, 25-, and 30-day-old females. The experimental procedure was repeated three times. A total of 270 pairs of adult males and females are required. The *dsVgR* and *dsGFP* treatment groups were handled identically, and the solution was replaced according to different treatment processes. The cDNA from these samples was prepared as previously described and used for ovary qRT–PCR assays. QRT-PCR was performed for each gene with three biological replicates and three technical replicates (Supplementary Materials).

### 2.5. RNAi for Oviposition Behavior

Ten newly emerged, unmated females were randomly selected, fed *dsVg4* (1 ng/μL, 300 μL), and paired with ten 7- to 12-day-old unmated males. A pair with a male and a female was placed in each rearing container. The *dsVgR*, *dsVg4* + *VgR*, blank control (sterile water) and *dsGFP* treatments were carried out as described for the *dsVg4* treatment. Each day at 8 am, *M. exotica* shoots were replaced when eggs were detected on the shoots. Change the solution in the rearing container once every 5 days. The replaced *M. exotica* shoots were stored separately after the number of eggs was counted. The number of nymphs was also counted after the eggs hatched. The preoviposition, fecundity, oviposition rhythm, egg hatchability, deformity rate of the eggs and nymphs, and female survival rate were assessed for 30 days. The experimental procedure was repeated five times. In total, 50 pairs of adult males and females are required.

### 2.6. Statistical Analysis

Data analysis was performed using SPSS 25.0 (IBM Corp., Armonk, New York, NY, USA) and GraphPad Prism 10 (GraphPad Software, San Diego, CA, USA). Data on the spatiotemporal expression of the *Vg* and *VgR* gene families were analyzed using Tukey’s honestly significant difference (HSD) multiple tests. Expression levels of the *Vg4* and *VgR* gene RNAi were analyzed using an independent samples *t* test. Number of mature eggs and egg length and width were analyzed using Tukey’s HSD. Data on the preoviposition, daily oviposition, number of eggs per female, egg hatchability, and deformity rates of eggs and nymphs were analyzed using one-way analysis of variance (ANOVA). Analysis of female survival rate between the interference group and the control group in the RNAi for oviposition behavior experiment used the log-rank (Mantel–Cox) test. The difference was considered to be statistically significant at the 5% level (*p* < 0.05).

## 3. Results

### 3.1. Spatiotemporal Expression of the Vg and VgR Genes

**Figure 1:**
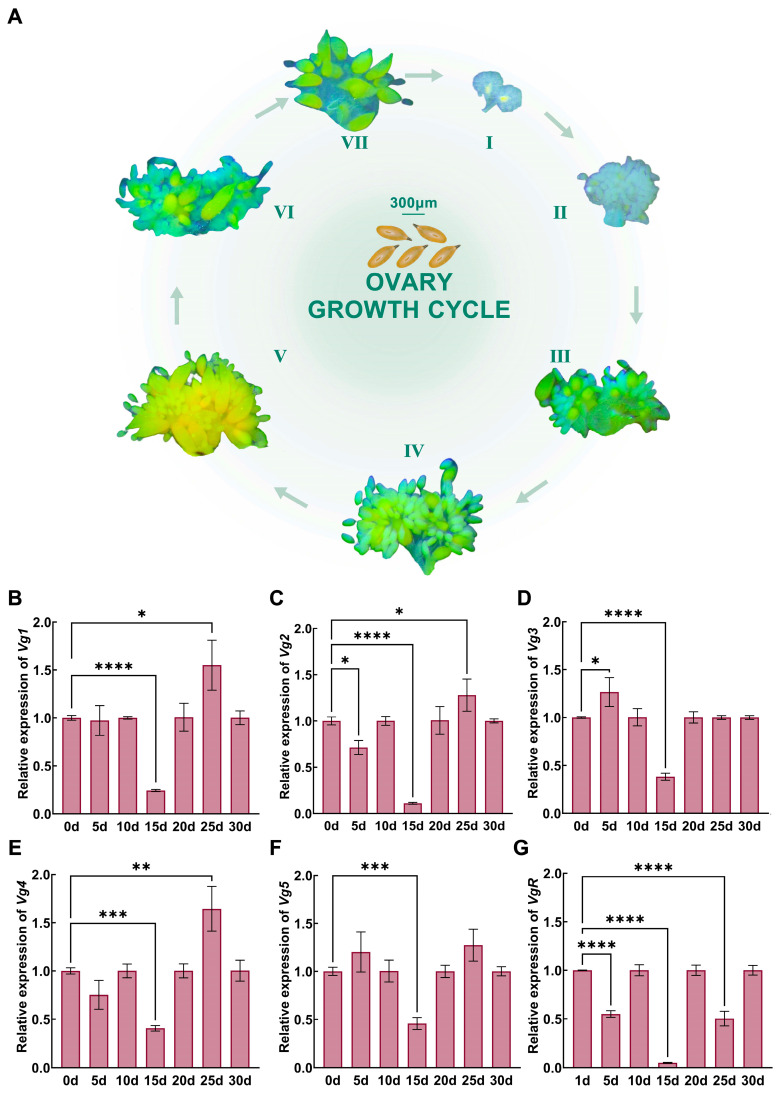
Morphological characteristics of ovarian development in *D. citri* females during the 1–30 d developmental stage, along with the spatiotemporal expression profiles of the *Vg* and *VgR* genes. (**A**) Ovarian development characteristics. (**B**–**G**) The relative expression levels of *Vg1*, *Vg2*, *Vg3*, *Vg4*, *Vg5*, and *VgR*, respectively, on the 1st, 5th, 10th, 15th, 20th, 25th, and 30th days. Data are shown as the mean  ±  SD, and *p* values are based on Tukey’s HSD multiple tests: **** *p*  <  0.0001; *** *p*  <  0.001; ** *p*  <  0.01; * *p*  <  0.05.

Female ovaries were dissected at the 1- to 30-day developmental stage, and the expression levels of ovarian *Vg* and *VgR* gene family members were detected using qRT–PCR technology (Figure 1; Supplementary Materials). The results indicate that during the 1- to 15-day developmental stage, oocyte development is characterized by a gradual increase in volume; during the 20- to 30-day stage, no significant differences in changes in the length or width of the oocytes were observed. The stage with the maximum length and width of the oocytes occurred at 25 days (length: 290.60 ± 2.10 µm; width: 119.46 ± 3.35 µm) (Figure 1A). *Vg* and *VgR* gene family members were expressed in the 1- to 30-day female adults. Compared with the expression levels of newly emerged females (1-day), the expression of *vitellogenin-1-like-1* (*Vg1*), *vitellogenin-1-like-2* (*Vg2*), *vitellogenin-2-like* (*Vg3*), *vitellogenin-3-like* (*Vg4*), *vitellogenin-like* (*Vg5*) and *vitellogenin receptor* (*VgR*) showed the most significant decrease at 15 days (*Vg1*, t = 47.68, *p* < 0.0001; *Vg2*, t = 34.70, *p* < 0.0001; *Vg3*, t = 28.42, *p* < 0.0001; *Vg4*, t = 24.37, *p* < 0.001; *Vg5*, t = 12.50, *p* < 0.001; *VgR*, t = 297.90, *p* < 0.0001; Figure 1B–G), whereas the expression of the five genes showed the most significant increase at 25 days (*Vg1*, t = 3.64, *p* < 0.05), 25 days (*Vg2*, t = 2.69, *p* < 0.05), 5 days (*Vg3*, t = 3.05, *p* < 0.05), and 25 days (*Vg4*, t = 4.75, *p* < 0.01), respectively (Figure 1B–E). However, there was no significant increase in *Vg5* and *VgR* in comparison in newly emerged females (Figure 1F,G).

### 3.2. Effects of Vg4 and VgR Gene RNAi on Ovarian Development

**Figure 2:**
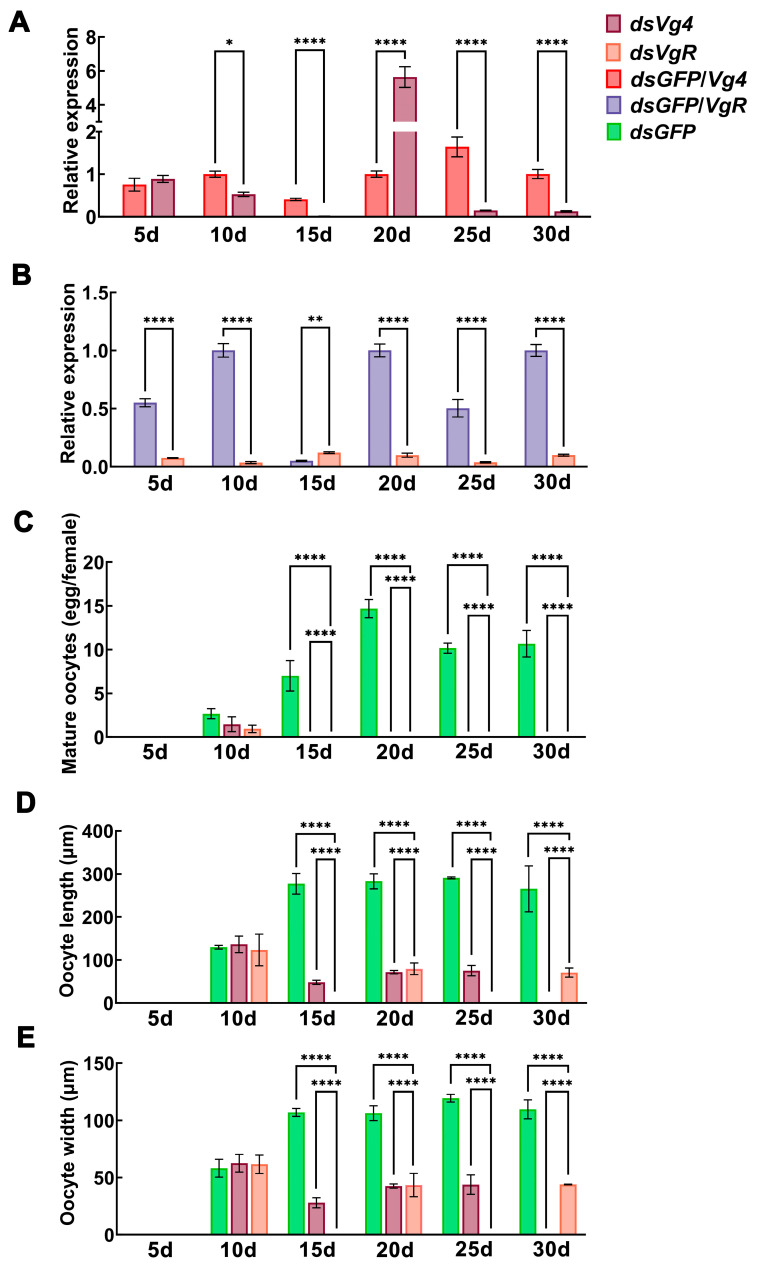
Interference effect of *dsVg4* and *dsVgR* on *Vg4* and *VgR* expression as well as ovary developmental and morphological characteristics in *D. citri* females during the 1–30 d developmental stage. (**A**,**B**) The relative expression levels of *Vg4* and *VgR* were analyzed using independent samples *t* test. (**C**–**E**) The number of mature oocytes, mature oocyte length, and mature oocyte width, its were analyzed using Tukey’s HSD, respectively. Data are shown as the mean  ±  SD; *p* values are based on the independent samples *t* test and Tukey’s HSD multiple tests: **** *p*  <  0.0001; ** *p*  <  0.01; * *p*  <  0.05.

Following continuous feeding on *dsVg4* and *dsVgR* during the 1- to 30-day ovarian development stage in *D. citri* females, both *Vg4* and *VgR* gene expression and ovarian development were effectively disrupted, with significant downregulation of *Vg4* and *VgR* gene expression in the females (Figure 2; Supplementary Materials). Compared with the *dsGFP* negative control, the *dsVg4* interference group exhibited the most significant decrease in *Vg4* gene expression at 15 days, with a relative expression reduction of 97.69% (*p* < 0.0001). However, at the 20-day ovarian development stage, the relative expression of the *Vg4* gene abnormally increased by 462.37% (*p* < 0.0001) (Figure 2A). In the *dsVgR* interference group, *VgR* gene expression decreased most significantly at 10 days, with a relative expression reduction of 96.59% (*p* < 0.0001). However, at the 15-day ovarian development stage, the relative expression of the *VgR* gene abnormally increased by 146.22% (*p* < 0.01) (Figure 2B).

An analysis of the mature oocyte number in the ovaries, as well as oocyte length and width, using Tukey’s HSD test revealed *dsVg4* and *dsVgR* interference groups. The mature oocyte number during the ovarian development stages from 15 to 30 days was 0.00 ± 0.00 per female (Figure 2C), which was significantly lower than that of the *dsGFP* negative control at 15 days (7.00 ± 1.73 mature oocytes per female; *dsVg4*, t = 7.00, *p* < 0.01; *dsVgR*, t = 7.00, *p* < 0.01), 20 days (14.67 ± 1.04 mature oocytes per female; *dsVg4*, t = 24.41, *p* < 0.001; *dsVgR*, t = 24.41, *p* < 0.001), 25 days (10.17 ± 0.58 mature oocytes per female; *dsVg4*, t = 30.50, *p* < 0.001; *dsVgR*, t = 30.50, *p* < 0.001), and 30 days (10.67 ± 1.53 mature oocytes per female; *dsVg4*, t = 12.09, *p* < 0.001; *dsVgR*, t = 12.09, *p* < 0.001). Interference with the expression of the *Vg4* and *VgR* genes effectively suppressed female mature oocyte formation. The average dimensions of the *dsVg4* interference group of oocytes were as follows: 15 days (length, 47.99 ± 4.89 µm; width, 28.02 ± 4.34 µm), 20 days (length, 71.81 ± 4.10 µm; width, 42.62 ± 1.77 µm), 25 days (length, 75.17 ± 12.13 µm; width, 43.79 ± 8.56 µm), and 30 days (length, 0.00 ± 0.00 µm; width, 0.00 ± 0.00 µm); the average dimensions of the *dsVgR* interference group of oocytes were 15 days (length, 0.00 ± 0.00 µm; width, 0.00 ± 0.00 µm), 20 days (length, 79.53 ± 13.57 µm; width, 43.46 ± 10.22 µm), 25 days (length, 0.00 ± 0.00 µm; width, 0.00 ± 0.00 µm), and 30 days (length, 70.64 ± 10.86 µm; width, 43.96 ± 0.29 µm) (Figure 2D,E). These values were significantly lower than those of the *dsGFP* negative control at 15 days (length 269.80 ± 28.47 µm; width 107.05 ± 3.54 µm), 20 days (length 282.89 ± 17.47 µm; width 106.38 ± 6.47 µm), 25 days (length 290.60 ± 2.10 µm; width 119.46 ± 3.35 µm), and 30 days (length 265.27 ± 53.22 µm; width 109.73 ± 8.32 µm) (Figure 2D,E). These results indicate that the *Vg4* and *VgR* genes are key genes for ovarian development and oocyte formation in *D. citri*.

### 3.3. Effects of Vg4 and VgR Gene RNAi on the Preoviposition Period and Oviposition Rhythms

**Figure 3:**
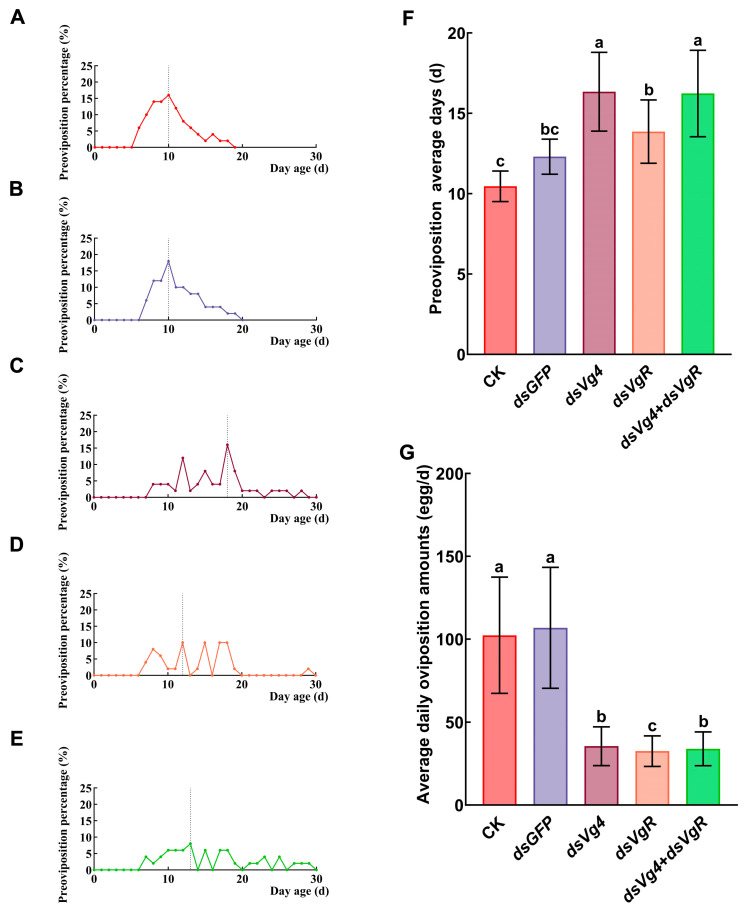
Comparison of the females’ first oviposition rate at different days of age, preoviposition period average days and average daily oviposition amounts in *D. citri* females across the 1–30 d developmental stage. (**A**–**E**) The females’ first oviposition rate at different days age of the control check (CK), *dsGFP* negative control, *dsVg4*-treated, *dsVgR*-treated and *dsVg4* + *dsVgR*-treated, respectively. (**F**,**G**) The preoviposition period average days and average daily oviposition amounts, respectively. Data are shown as the means  ±  SDs, and different lowercase letters indicate significant differences based on the ANOVA (*p* < 0.05).

During the 1- to 30-day ovarian development stage, we observed the effects of continuous feeding on *dsVg4*, *dsVgR*, and *dsVg4* + *dsVgR* during the preoviposition period and oviposition rhythm in *D. citri* females (Figure 3). The longest average preoviposition period was observed in the *dsVg4* interference group; females required 7 days of feeding posteclosion to reach the oviposition stage, with an average preoviposition period of 16.34 ± 2.45 days. We checked the 50-female oviposition every day (total eggs: 1065) and found average daily oviposition amounts of 35.50 ± 11.67 eggs per day (Figure 3C,F,G). The lowest average daily oviposition amount was observed in the *dsVgR* interference group; females required 6 days of feeding posteclosion to reach the oviposition stage, with an average preoviposition period of 13.86 ± 1.97 days. We checked the 50-female oviposition every day (total eggs 976) and found average daily oviposition amounts of 32.53 ± 9.21 eggs per day (Figure 3D,F,G).

**Figure 4:**
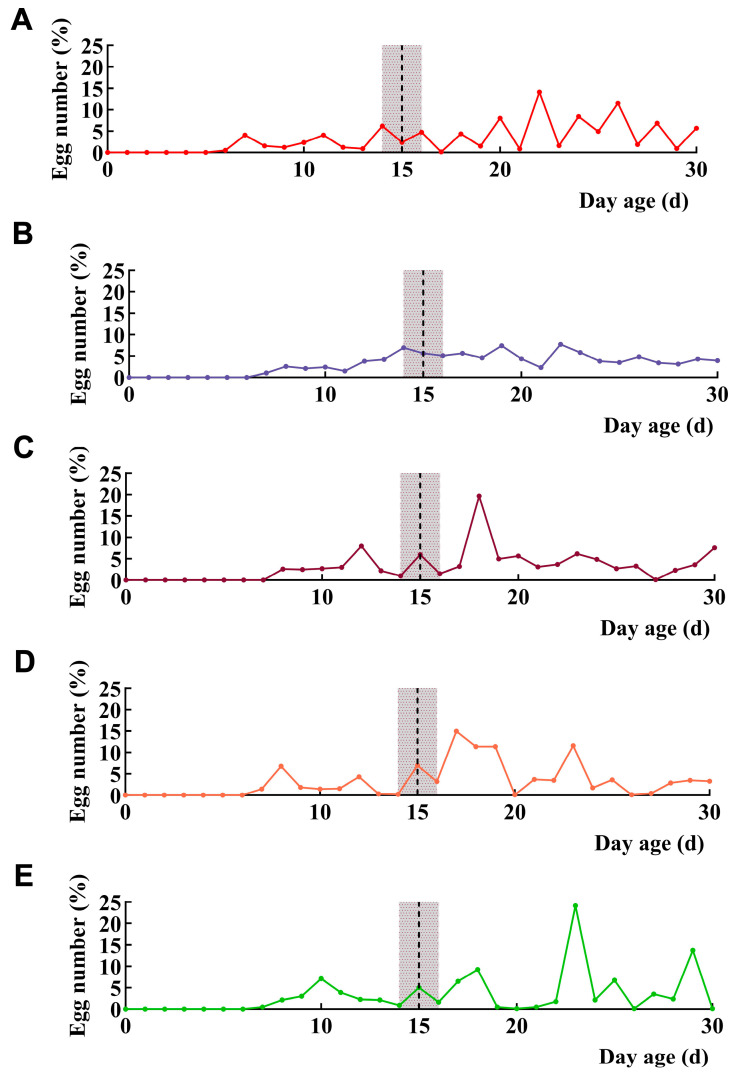
Oviposition daily rhythm of *D. citri* females across 1–30 d developmental stage. (**A**–**E**) The daily oviposition rhythms of control check (CK), *dsGFP* negative control, *dsVg4*-treated, *dsVgR*-treated and *dsVg4* + *dsVgR*-treated, respectively.

The average preoviposition period of the interference group was generally greater than that of the control group, while the average daily oviposition amount was significantly lower than that of the control group. Comparisons of the oviposition rhythm data revealed that the female oviposition rhythm in the interference group significantly fluctuated, with weaker persistence of oviposition activity in the interference group than in the control group. The female oviposition amount in the *dsVg4*, *dsVgR* and *dsVg4* + *dsVgR* interference groups during the 15- to 30-day ovarian development stage accounted for 78.15%, 82.17% and 78.13% of the total oviposition amount, respectively (Figure 4C–E). The females in the CK and *dsGFP* groups exhibited highly sustained oviposition activity, with periodic fluctuations in daily peaks, though overall showing an increasing-to-decreasing trend; however, overall, the oviposition amount contributed 77.92% and 75.44% of the total oviposition amount during the 15- to 30-day ovarian development stage, respectively (Figure 4A,B).

### 3.4. Effects of Vg4 and VgR Gene RNAi on Female Reproductive Capacity and Survival Rate

**Figure 5:**
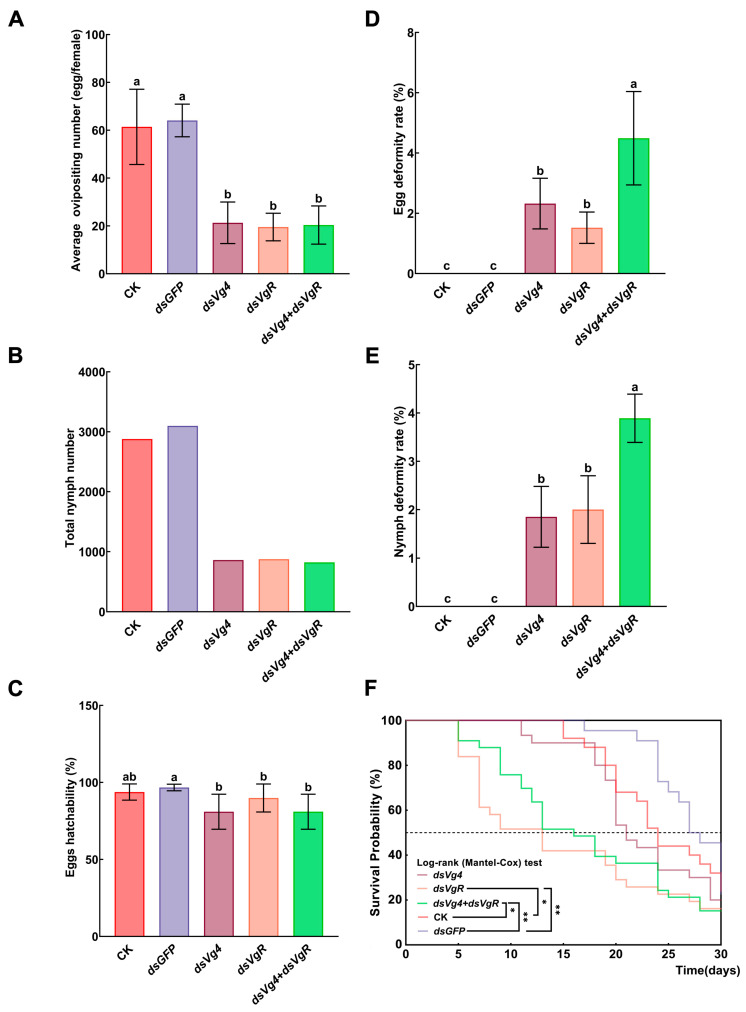
Comparison of reproductive capacity and survival rate in *D. citri* females across 1–30 d developmental stages. (**A**–**F**) The average oviposition number, total nymph number, egg hatchability, egg deformity rate, nymph deformity rate, and survival probability, respectively. (**A**,**C**–**E**) Results were analyzed using ANOVA and are shown as the means  ±  SDs; different lowercase letters indicate significant differences based on the ANOVA (*p* < 0.05). (**F**) Results analyzed using log-rank (Mantel–Cox) test: ** *p*  <  0.01; * *p*  <  0.05.

A comparison of the reproductive capacity and survival rates of *D. citri* females in the 1- to 30-day ovarian development stage between the interference and control groups revealed that the total oviposition amount per female in the *dsVg4*, *dsVgR*, and *dsVg4* + *dsVgR* interference groups was significantly lower than that in the control group. Females in the *dsVgR* interference group presented the lowest average total oviposition amount per female (19.52 ± 5.76 eggs per female; total eggs 976), whereas those in the *dsVg4* + *dsVgR* interference group hatched the fewest total nymphs (hatched nymphs 670) (Figure 5A,B). The lowest egg hatching rate was observed in the *dsVg4* interference group; all the treatment groups presented egg hatching rates above 80%, and the majority of the eggs successfully hatched (Figure 5C). The highest rates of egg and nymph deformities were observed in the *dsVg4* + *dsVgR* interference group, with an egg deformity rate of 4.49 ± 1.55% and a nymph deformity rate of 3.89 ± 0.50% (Figure 5D,E). A comparison of the survival rate data from the log-rank (Mantel–Cox) test revealed that compared with those in the other treatment groups, the lifespan of females in the *dsVgR* and *dsVg4* + *dsVgR* interference groups was significantly shorter during the 1- to 30-day ovarian development stage. In the *DsVgR* interference group compared with CK, the median survival (time taken to reach a survival of 50%) of CK females was 24 (95% CI of ratio: 1.02–3.34) days compared to 13 (95% CI of ratio: 0.30 to 0.98) days for *dsVgR* interference group females; this translates to a 45.83% decrease in longevity in *dsVgR* interference group females. In the *dsVgR* interference group compared with the *dsGFP* negative control, the median survival (time taken to reach a survival of 50%) of *dsGFP* females was 27.5 (95% CI of ratio: 1.15–3.90) days compared to 13 (95% CI of ratio: 0.26 to 0.87) days for *dsVgR* interference group females; this translates to a 52.73% decrease in longevity in *dsVgR* interference group females (Figure 5F). In the *DsVg4* + *dsVgR* interference group compared with CK, the median survival (time taken to reach a survival of 50%) of CK females was 24 (95% CI of ratio: 0.84–2.69) days compared to 16 (95% CI of ratio: 0.37 to 1.19) days for *dsVg4* + *dsVgR* interference group females; this translates to a 33.33% decrease in longevity in *dsVg4* + *dsVgR* interference group females. In the *dsVg4* + *dsVgR* interference group compared with the *dsGFP* negative control, the median survival (time taken to reach a survival of 50%) of *dsGFP* females was 27.5 (95% CI of ratio: 0.94–3.14) days compared to 16 (95% CI of ratio: 0.32 to 1.06) days for *dsVg4* + *dsVgR* interference group females; this translates to a 41.82% decrease in longevity in *DsVgR* interference group females (Figure 5F).

## 4. Discussion

This study investigated the spatiotemporal expression mechanisms of the *Vg4* and *VgR* genes in *D. citri* females during ovarian development and oviposition behavior. Both the *Vg4* and *VgR* genes govern ovarian development and control oviposition behavior progression. A spatiotemporal expression study using qRT–PCR and ovarian dissection revealed that 15 days was the time point at which the lowest expression levels of the *Vg* and *VgR* gene families occurred, which coincided with the period of rapid oocyte volume expansion. *D. citri* females go through developmental stages from 1 to 15 days, in which expression of the *Vg* and *VgR* genes primarily promotes oocyte maturation, and from 15 to 30 days, in which expression of these genes primarily promotes the progression of oviposition behavior. It is worth noting that the relative expression of the *VgR* and *Vg4* genes was significantly elevated at the 15- and 20-day developmental stages (Figure 2). This phenomenon may be attributed to the regulation of cellular immunity by dsRNA. Previous studies have shown that dsRNA-degrading enzymes (dsRNases) are key factors in reducing various insect species the efficiency of RNA interference and may lead to increased gene expression, but the related molecular mechanism is not clear; further research is needed [41,42]. Therefore, the expression of egg-laying-related gene pathways in the citrus psyllid at the 15- and 20-day developmental stages warrants further investigation. Comparisons of oviposition rhythm data revealed that during the 15- to 30-day developmental stage, females in the interference and control groups accounted for 75–85% of the total oviposition amount, respectively. Oocyte maturation predominantly occurred after 15 days of ovarian development. *D. citri* female oocyte development and oviposition behavior are phased, reflecting the staged expression of the *Vg* and *VgR* genes. Their peak expression time is strongly linked to ovarian developmental phases, making them useful molecular markers for analyzing *D. citri* female reproductive patterns [43]. However, research into the gene networks underlying the staged expression of the *Vg* and *VgR* genes in *D. citri* females is lacking, as well as into how these genes interact with other hormone signaling pathways or regulatory networks to regulate these processes, requiring further investigation.

According to previous studies, *Vg* and *VgR* genes show low expression during the egg or larval stages in some insects, with significant upregulation occurring during the pupal stage. Using *Tomicus yunnanensis* as an example, the peak expression levels of the *Vg* and *VgR* genes in *T. yunnanensis* females can exceed pupal levels by more than twice [44]. This finding demonstrates that the high expression phenomenon of the *Vg* and *VgR* genes does not persist throughout the life cycle of the insect. Instead, it has a three-phase rhythm: expression during adulthood, a peak specific to ovarian development, and a quick decrease following yolk filling [45,46]. The expression levels of the *Vg* and *VgR* genes in *D. citri* females decrease rapidly at the 15-day developmental stage, which could be attributable to the particular suppression of *Vg* and *VgR* gene expression by upstream or downstream regulatory genes after the peak accumulation of yolk in the ovaries. However, more research is needed on the underlying molecular pathways that govern this phenomenon.

Recent research suggests that essential gene pathways controlling insect oviposition involve several hormones signaling and dietary regulation networks [47,48,49,50]. Among them, the juvenile hormone (JH) pathway may be one of the primary regulatory systems that controls the expression of *Vg* and *VgR* genes [51]. The JH pathway influences insect oviposition by activating Vg protein synthesis via the methoprene-tolerant/Taiman (Met/Tai) receptor complex, which regulates lipocyte polyploidization and follicle cell channel opening, promoting Vg protein entry into the oocyte [52]. Using *Diaphorina citri* (Kuwayama) as an example, disrupting female *JH* gene expression resulted in impaired ovarian development and a significant reduction in reproductive capacity in females (interference group: 136.61 eggs per female; CK: 236.29 eggs per female), demonstrating the certain critical role of JH in female oviposition [52]. *Methoprene-tolerant* (*Met*), *Juvenile hormone* (*JH*), *Taiman* (*Tai*), *Vitellogenin* (*Vg*), and *Vitellogenin receptor* (*VgR*) are some key genes for regulating insect oviposition. The *JH*, *Met*, and *Tai* genes are upstream regulators of *Vg* and *VgR* gene expression and may serve as ideal RNAi targets for *D. citri* regulation research, but more research is needed to verify this [53,54,55,56]. The JH pathway regulates *Vg* and *VgR* gene expression, providing vital insights into the relationships and regulatory processes among the *Vg*, *VgR*, *JH*, *Met*, and *Tai* gene families in female *D. citri*. In future studies, nanomaterials (chitosan) or pesticides (metaflumizone) at different concentrations could be used as co-delivery systems for dsRNA to enhance the interference effect of dsRNA targeting JH pathway genes, thereby further elucidating the mechanisms by which the JH pathway regulates oviposition in *D. citri* [57,58].

## 5. Conclusions

In conclusion, during the 1- to 15-day developmental stage, the expression of the *Vg* and *VgR* genes predominantly promotes oocyte maturation; during the 15- to 30-day developmental stage, the expression of these genes mostly promotes oviposition behavior progression within the ovaries. RNAi interference tests revealed that the *Vg4* and *VgR* genes play critical roles in ovarian development and oviposition behavior in *D. citri* females. Future studies will focus on three main areas. First, we will perform in-depth studies on the pathway expression mechanisms of the *Vg* and *VgR* genes, with a focus on their connections with the JH signaling pathway. Second, we will construct *dsVg4*- and *dsVgR*-related bacterial vectors to improve their stability. Third, using the *Vg* and *VgR* genes as key network nodes, we will investigate the pathway expression mechanisms that control genes linked with oviposition behavior.

## Figures and Tables

**Table 1:** Oligonucleotide primer pairs used in this study.

Gene	Primer Name	Sequences of Primers (5′ → 3′)	Application
*Vg1*	Vg1 F1	TACGCTGGATTTGCTT	qRT-PCR
	Vg1 R1	TTGACGGATTTGTGGT	
*Vg2*	Vg2 F1	CCACCTACTCCTTGTCC	qRT-PCR
	Vg2 R1	ATCGTTTGGCGTCAGC	
*Vg3*	Vg3 F1	TACGGAGAATCCAGCAC	qRT-PCR
	Vg3 R1	GGCGTAGGAGGTAAGG	
*Vg4*	Vg4 F1	ATGGCCATGAAACAATGGAT	PCR
	Vg4 R1	AAGACGTTGGAAGTTGGTGG	
	Vg4T7 F1	TAATACGACTCACTATAGGG ATGGCCATGAAACAATGGAT	dsRNA synthesis
	Vg4T7 R1	TAATACGACTCACTATAGGG AAGACGTTGGAAGTTGGTGG	
	Vg4 F2	GAAAGATACATCCCATACT	qRT-PCR; RNAi fragment qRT-PCR
	Vg4 R2	GAATACAGCAGCACAAC	
*Vg5*	Vg5 F1	AAGACACCGTCACTGGA	qRT-PCR
	Vg5 R1	GGAAGTCCGAAGTGGTA	
*VgR*	VgR F1	ACGCTCACATGGGACCTAAC	PCR
	VgR R1	GACGTCCAATACATTCGCCT	
	VgRT7 F1	TAATACGACTCACTATAGGG ACGCTCACATGGGACCTAAC	dsRNA synthesis
	VgRT7 R1	TAATACGACTCACTATAGGG GACGTCCAATACATTCGCCT	
	VgR F2	TGATGGCAATGATGAC	qRT-PCR; RNAi fragment qRT-PCR
	VgR R2	GGCTGGGTAGTGTAGAA	
*GFP*	GFP F1	ATGGTGAGCAAGGGCGAGGAG	PCR
	GFP R1	CTTGTACAGCTCGTCCATGCCG	
	GFPT7 F1	TAATACGACTCACTATAGGG ATGGTGAGCAAGGGCGAGGAG	dsRNA synthesis
	GFPT7 R1	TAATACGACTCACTATAGGG CTTGTACAGCTCGTCCATGCCG	
*Actin*	Actin F	TGTGACGAAGAAGTTGCTGC	qRT-PCR
	Actin R	TGGGGTATTTCAGGGTCAGG	
*EF1a*	EF1a F	GCCAACCTCACCACTG	qRT-PCR
	EF1a R	GCGACGAAACCACGAC	

The T7 RNA polymerase promoter is underlined.

## Data Availability

The original contributions presented in this study are included in the article/Supplementary Materials. Further inquiries can be directed to the corresponding author.

## Supplementary Materials

The following supporting information can be downloaded at https://www.mdpi.com/article/10.3390/insects17060562/s1. Figure S1: *Vg1* gene qRT-PCR amplification, melting curve and standard curve; Figure S2: *Vg2* gene qRT-PCR amplification, melting curve and standard curve; Figure S3: *Vg3* gene qRT-PCR amplification, melting curve and standard curve; Figure S4: *Vg4* gene qRT-PCR amplification, melting curve and standard curve; Figure S5: *Vg5* gene qRT-PCR amplification, melting curve and standard curve; Figure S6: *VgR* gene qRT-PCR amplification, melting curve and standard curve; Figure S7: *Actin* gene qRT-PCR amplification, melting curve and standard curve; Figure S8: *EF1a* gene qRT-PCR amplification, melting curve and standard curve; Figure S9: Ovaries at the newly eclosion stage (0d); Figure S10: Ovaries at the 5-day ovarian development stage; Figure S11: Ovaries at the 10-day ovarian development stage; Figure S12: Ovaries at the 15-day ovarian development stage; Figure S13: Ovaries at the 20-day ovarian development stage; Figure S14: Ovaries at the 25-day ovarian development stage; Figure S15: Ovaries at the 30-day ovarian development stage; Figure S16: *DsVg4*, *dsVgR*, and *dsGFP* were absorbed by tender *M. odorifera* shoots for six days, and gel electrophoresis was used to detect the persistence of *dsVg4*, *dsVgR*, and *dsGFP* after each day. The gel electrophoresis bands of *dsVg4* were relatively clear at 1–6 d; the gel electrophoresis bands of *dsVgR* were relatively clear at 1–2 d, but blurry at 3–6 d; the gel electrophoresis bands of *dsGFP* were relatively clear at 1–6 d. The *dsVg4*-1, 2, 3, 4, 5, 6/*dsVgR*-1, 2, 3, 4, 5, 6/*dsGFP*-1, 2, 3, 4, 5, 6 represent the six replicates for each treatment; Figure S17: After *M. odorifera* shoots total RNA of absorbing *dsVg4* and *dsVgR* were extracted, the sequencing results of *Vg4* and *VgR* gene interference fragments were cloned using cDNA as template. (A) Base sequence and base feasibility of *Vg4* gene, sequence alignment between the *Vg4* sequencing sequence and the original sequence. (B) Base sequence and base feasibility of *VgR* gene, sequence alignment between the *VgR* sequencing sequence and the original sequence; Table S1: Gene qRT-PCR CT value; Table S2: Gene RNAi qRT-PCR CT value.
