# Supplementary material for: Vitellogenin-3-like and Vitellogenin receptor Genes Involved in the Regulation of Ovarian Development and Oviposition in Diaphorina citri
Source: Insects. 2026 May 29;17(6):562. doi: 10.3390/insects17060562 (PMC13299296; doi:10.3390/insects17060562)
Supplement: Supplementary file 1 [file insects-17-00562-s001.zip › insects-4283092-supplementary.pdf]

Supplementary Materials

1. DsRNA synthesis

The RNA synthesis system is as follows:

**Name of ingredients**	**Dosage**
RiboMAX™ Express T7 2X Buffer*	10.0 μL
Transcriptional template	6.0 μL
DEPC water	2.0 μL
Enzyme Mix, T7 Express	2.0 μL
Total volume	20.0 μL

In vitro transcription, react at 37℃ for 30 min; after holding at 70℃ for 10 minutes, hold at room temperature for 20 minutes; after standing at 37℃ for 30 minutes, place on ice for 5 minutes; centrifuge for 10 minutes, annealed to obtain dsRNA.

2. Full-length sequences of *Vitellogenin* genes and *Vitellogenin receptor* gene (qRT-PCR sequences: red label)

LOC103523873 *Vitellogenin-1-like-1* [*Diaphorina citri* (Asian citrus psyllid)]

Gene ID: 103523873, updated on 15-Jul-2019 1858 bp *Vg1*

>RNA-XM_008488883.3, gene id: LOC103523873, gene sequence

ATGGCTGTTGCCGCCACTGCTCAAGTTCCCCAACTTTCCGAAATGAAC
TTCATGAGATCTGAAAAGGAAGACCCTGCCATGCAAATCGCCGCTGA
AGCCATGTGGGGAGAGAATGCTCAATCTGGAGCTAAAATCAGCATCA
AGGCTAAACTCGAACAATCCGAACAACGCAAACAATACATTGCCAAC
CACCCACAAGCTGAACAATGCAGAAAACAAATGGAACAAAGAGACT
ATGCTCTCAATGCTTGCCGCAACGTCACTGCTAGATCCAATGCTCTTG
ATGAATACTCTCTCACCATCAAATACGAAAAGATCCCACAAAAGCTG

ATGAACGCAACCTACCAACTCTACAGAATCGCCCGTTACGCTGGATTT
GCTTACAATTCTGAAAATGTTGTTGCTGTTTCCAACCAAGCCGACCAA
TTGAAAGTCAGAGTGAACATTGCTGAAGACCACAAATCCGTCAATGTT
TCCATTGAAGCCCCACACGCCAACTCTCAATTCAACAACCTCCCACTT
TCCAACATGGCTAAACACATCCTCATCCAAAACGCTCAATACGATGTA
GACCAAAGAGTAGGATACGCTGCATTCAATGGCCAATACAACCCTGT
GTGTGTTGCTGATGGATCATCCGGTCAAACCTTCGACAACAAGACCTA
CCCACTGAACCTTGAAAAAGACTCTTGGTACGTGCTCATGACTTCTGC
TCCCAAACAAAGAAAAGACAACAGAGTTGACTACAAAACCCAAAGA
CAAGAAAACGTCACCATCCTGGTCAAACAATCCGGAGAAAACAAAA
AGGAACTCAAAATCGTCTTGAACAACGGAGAACATGTTATTGACATG
CAACCCTCTTCCTCCAACAACGATGGTGCCAACGCCAAAATCCAAGT
CAACAAGAAAGACCAAAGAGCCTCCAAGAACAGCGTTACTGAAGTC
ACCGACAACCAAAACCAAAGAATTGCTCAGATCTACGCTCTGCCCAG
TGGAGAAGTCATCGTCAACATGCCCAACCATGGACTTAGACTCAACT
ACGATGGAAGCCGCGTTCAAGTTCAAGCCACCGACCGTTTCAGAGAC
GGAGTTCGTGGACTCTGTGGATCTTTCACTGGAGAAAAAGCCACCGAC
TTCATCACCCCAAGAAACTGCATCGTCAGAGAAGCCAAAGACTTCGTT
GCCACCTACTCCTTGTCCTCTAACAACAGAGACAACTCCAGACTCTCT
AACCAAGACTTCTGTGCCCCCAAGAGACAAGTTCAATTCCAACAAGT
CATCAACGAAAAGAACATTGGACGTTGGGCTGACGCCAAACGATTGA
ATCTGCAAGGACTCTGGGGATTTTTCAACAAGAATGATGATGATGATA
ACAGCAGCAACAGCCAGAACAACTCTGGAAACAAGAACAACAGACG
TAGCCCCAGACATGGAAACAACAGCCAAAACAGTAACGAAAACGAC
AGCAACAACCAAAACAGCAATGAAAACAACAGAAACAACCAAGGC
AGCGACGAGAACAACAAGAACAACCAACAACGTGGAAGTTCCAGCC
ACCGCCTCATGGTCGTTGAACAAGGAAACCAACTGTGCTTCAGTACCA
AGGCCATGCCCCAATGTAACCAAGGATACCGTGCTGAAAACACCGTT
GAAAAGAAGGTACGTGATCACTAA
>RNA-XM_008488883.3, gene id: LOC103523873, amino acid sequence

MAVAATAQVPQLSEMNFMRSEKEDPAMQIAAEAMWGENAQSGAKISIK
AKLEQSEQRKQYIANHPQAEQCRKQMEQRDYALNACRNVTARSNALDE
YSLTIKYEKIPQKLMNATYQLYRIARYAGFAYNSENVVAVSNQADQLKVR
VNIAEDHKSVNVSIEAPHANSQFNNLPLSNMAKHILIQNAQYDVDQRVG
YAAFNGQYNPVCVADGSSGQTFDNKTYPLNLEKDSWYVLMTSAPKQRKD
NRVDYKTQRQENVTILVKQSGENKKELKIVLNNGEHVIDMQPSSSNNDGA
NAKIQVNKKDQRASKNSVTEVTDNQNQRIAQIYALPSGEVIVNMPNHGL
RLNYDGSRVQVQATDRFRDGVRGLCGSFTGEKATDFITPRNCIVREAKDFV
ATYSLSSNNRDNSRLSNQDFCAPKRQVQFQQVINEKNIGRWADAKRLNL
QGLWGFFNKNDDDDNSSNSQNNSGNKNNRRSPRHGNNSQNSNENDSN
NQNSNENNRNNQGSDENNKNNQQRGSSSHRLMVVEQGNQLCFSTKAM
PQCNQGYRAENTVEKKVRDH

LOC103523874 ***Vitellogenin-1-like-2*** **[*Diaphorina citri* (Asian citrus psyllid)]**

Gene ID: 103523874, updated on 15-Jul-2019 1116 bp *Vg2*

>RNA-XM_008488884.1, gene id: LOC103523874, gene sequence

ATGCCCAACCATGGACTTAGACTCAACTACGATGGAAGCCGCGTTCA
AGTTCAAGCCACCGACCGTTTCAGAGACGGAGTTCGTGGACTCTGTGG
ATCTTTCACTGGAGAAAAAGCCACCGACTTCATCACCCCAAGAAACT
GCATCGTCAGAGAAGCCAAAGACTTCGTTGCCACCTACTCCTTGTCCT
CTAACAACAGAGACAACTCCAGACTCTCTAACCAAGACTTCTGTGCCC
CCAAGAGACAAGTTCAATTCCAACAAGTCATCAACGAAAAGAACATT
GGACGTTGGGCTGACGCCAAACGATTGAATCTGCAAGGACTCTGGGG
ATTTTTCAACAAGAATGATGATGATGATGATAACAGCAGCAACAGCC
AGAACAACTCTGGAAACAAGAACAACAGACGTAGCCCCAGACATGG
AAACAACAGCCAAAACAGTAACGAAAACGACAGCAACAACCAAAA
CAGCAATGAAAACAACAGAAACAACCAAGGCAGCGACGAGAACAA
CAAGAACAACCAACAACGTGGAAGTTCCAGCCACCGCCTCATGGTCG
TTGAACAAGGAAACCAACTGTGCTTCAGTACCAAGGCCATGCCCCAA
TGTAACCAAGGATACCGTGCTGAAAACACCGTTGAAAAGAAGATCGA
TGCCCACTGTGTTCAAGACGGACAGTTAGCCAGACAATGGAAAGAAC
AAGCTCGCAGAGGTGAACACATCGCTGCCATGCAAAAGAAAAACCC
CAACAAGACCATCACCGTTGAAGTACCAACCAAATGTGTTGCTGCCTA
A

>RNA-XM_008488884.1, gene id: LOC103523874, amino acid sequence

MPNHGLRLNYDGSRVQVQATDRFRDGVRGLCGSFTGEKATDFITPRNCIV
REAKDFVATYSLSSNNRDNSRLSNQDFCAPKRQVQFQQVINEKNIGRWAD
AKRLNLQGLWGFFNKNDDDDDNSSNSQNNSGNKNNRRSPRHGNNSQN
SNENDSNNQNSNENNRNNQGSDENNKNNQQRGSSSHRLMVVEQGNQL
CFSTKAMPQCNQGYRAENTVEKKIDAHCVQDGQLARQWKEQARRGEHI
AAMQKKNPNKTITVEVPTKCVAA

LOC103513507 ***Vitellogenin-2-like*** **[*Diaphorina citri* (Asian citrus psyllid)]**

Gene ID: 103513507, updated on 15-Jul-2019 1197 bp *Vg3*

>RNA-XM_026826679.1, gene id: LOC103513507, gene sequence

ATGAACAAATATAATAAACTACTCAGTGAAAATCCAAGACTCCTTTA
CATCAAGGGGGTTCAAATCCCGACCAAGAATGATGGAATTGTATCCT
ACAATATGACAACAGTTAAGAATCTGCAAGTCAGCCAAAACCTGCCA
ACCTGGGAACTGAACATCATCAAGAGTATTGTCAGTCAACTCCAAGTT
GACACCAGAGCCGAAAATGAAGTCAGCTCGCGTCTCAACCAGAAACC
CAAGAACGGCAAACCTTTCGGAACCTTCAAGACCATGGAAGACACCG
TCACTGGAGAATGTGAAACCTTGTACGACATCAAACCTCTGCAACAA
AATCGAATACCACTTCGGACTTCCCAAGTTGCCTTCAAGGTCGAACCT
TTGAACAAGAACATGGAAGAGAAAATCTTCATCGCCACCCAACACCC
ATACACCACCATCCAAAACATCCTCAACTTGACCCCAGCTGAAAAAT
CACAAAACACCAAGGACATCAGCGTCAGAAAAACTAGAGAGTGGAC
CCAAACTTACGGAGAATCCAGCACTGGAGTTGCCATGAAAGTTCACT
ACAACGGTGAAGACAAAGCTGCTGACCTCGCATCCTTCTTGAGATCA
ATGCAAGGTCATGACCTTACCTCCTACGCCTTGTTGAACAACTTCAAC
GTTGAAATCAACAGACACAGATTCATTGTTTCCCACGATGCCCAAAAA
TCATCTGCCAAGGCTATTAGATTCTACGCTAGCTACAGAAACAACGAA
GAAAGCCGACTGAGAAACGCTGACAAAAACAACAATGACAATGCTA
ACAACAGTCCTCTTGAAGCTAGAGCTTCATCCGCTATTCCATCCACCC
CATCCAAAAATGGAGCTGAAAAACGCCAAGAAGAACTCTTACACAA
AGCTACTTCTGGAATCAAAGATGCTGCCGCTACTGTAGTTGAAATCCT
GACCGAATTCGAAGGAAACAACAACGCTCAATATGTTTTGACTGCTG
CCATTGCCAAGAGCCCAGTTGACAAGAATTCCAAATTCCTGTTCTTCT
ACCATGCTTCTCCAGCTCAATCATCCAAATTTGAAATGGCTGTTGCCG
CCACTGCTCAAGTTCCCCAACTTTCCGAAATGAACTTCATGAGATCTG
AAAAGGAACTATATAACATCCACTGTCATTTCTGTCATTTAAAGAGGT
GTGGGGA

>RNA-XM_026826679.1, gene id: LOC103513507, amino acid sequence

MNKYNKLLSENPRLLYIKGVQIPTKNDGIVSYNMTTVKNLQVSQNLPTWE
LNIIKSIVSQLQVDTRAENEVSSRLNQKPKNGKPFGTFKTMEDTVTGECETL
YDIKPLQQNRIPLRTSQVAFKVEPLNKNMEEKIFIATQHPYTTIQNILNLTP
AEKSQNTKDISVRKTREWTQTYGESSTGVAMKVHYNGEDKAADLASFLRS
MQGHDLTSYALLNNFNVEINRHRFIVSHDAQKSSAKAIRFYASYRNNEESR
LRNADKNNNDNANNSPLEARASSAIPSTPSKNGAEKRQEELLHKATSGIK
DAAATVVEILTEFEGNNNAQYVLTAAIAKSPVDKNSKFLFFYHASPAQSSK
FEMAVAATAQVPQLSEMNFMRSEKELYNIHCHFCHLKRCGDI

LOC103523199 *Vitellogenin-3-like* [*Diaphorina citri* (Asian citrus psyllid)]

Gene ID: 103523199, updated on 15-Jul-2019 4808 bp *Vg4*

>RNA-XM_026832896.1, gene id: LOC103523199, gene sequence

ATGAGCTCCGAACAAGGAAAGAACAGAAGAATCAGATCCCGTCGTTC
TGTCATCAGAAACTCAAACGAAGACAACAACGAAAACAAGAACAAC
AAGAGACACAGTGACAATAACAGAGACAATGACTACAATGATGATG
ACCAAAAGAATCACCAAAACTCTGGATCCCACAACAACAACAACAA
CAACAACAACAACAACAACAACAATGACAACGGAAACAACAACTAC
AACAACAACGCCCGTGCATGGAAACAAGGACAAGTCTATGAATACC
AAATCCAAGGCCGCACCCTAGCTGCTCTTCATGATGTAGCTGACCAAT
ACACCGGAACCATCATCAAAGCCACCCTCAAGGTTCAACCCAGAAAT
CAAGACTCAGTCTTGGCTTGGGTCACCAACGCTAGACACTCTGATGTT
CACGCCAACCTAACCAATGGCTGGAACCAAGAGATCCCAGACAAATA
CCTCAACTACCAAAACTGGCAACTCAGTGACAAACCATTCGCCATCC
AATTCAAGAATGGAGTTGTTAAGAATCTGCAAGTCAGCCAAAACCTG
CCAACCTGGGAACTGAACATCATCAAGAGTATTGTCAGTCAACTCCA
AGTTGACACCAGAGCCGAAAATGAAGTCAGCTCGCGTCTCAACCAGA
AACCCAAGAACGGCAAACCTTTCGGAACCTTCAAGACCATGGAAGAC
ACCGTCACTGGAGAATGTGAAACCTTGTACGACATCAAACCTCTGCA
ACAAGTTGAACAACAAAACAAACCTCAACTCGCCCCCATGCCAAACC
TCAAGGGATCAAACGGAGACCTTATTGACATCATCAAGACCAAGAAC
TTCAGTCGTTGTGACTCCAGAATCGAATACCACTTCGGACTTCCCGGA
AGCAACGATGTTGAACCATCAAGCAACCAAGTTCTTAACTTCTTGTCC
AGATCTTCAACCAGCAGAGTCATCATTGCTGGAGACCTGAGCCACTAC
ACCATCCAATCCTCAGTCACCACCGACAAAATTGTCATCAGTCCCGAA
CTCTACAACAAACAAAAGGGAATGGTTGTCAGTCGCCTGAACGTTAC
TCTGTCCAACGTCCACTCTGCTGCTCAAAACAATGCCCCAGCTCTCCC
ATCCAACGTCAACAAAGTTGATGATTTGATCTACGAATACAACCCAGC
TTCCCCTGACAACGAATCTGCCCAAAACAACAACGGCAAGAACAACC
ACCACAACGACGATGACTCATCCTCATCTTCTTCTTCAAGTTCTTCCAG
CTCCTCTTCTTCATCCTCCAGCAGTTCTGACAGTTCCTCCTCTTCTTCCT
CGTCCTCCTCCTCAGACAGCTCATCATCTTCTGACAGTTCCAGCTCTTC
CTCTGACAGCTCAAACTCTTCCTCCAACAGTTCCGAAAGTGACGAGAG
ACAAAACAACAAGAGACGCAATAACAACAGCGAAGAAAACAACAA
GAAGAACCACAACAACAGAAGCAACGAAAGTAGAAACAACAAAAA
CCACAATGGCAACAGCAATGAAAACAGAAACAAGAACAACGAAGAC
AACGACTACACCATGAGCTCCGAACAAGGAAAGAACAGAAGAATCA
GATCCCGTCGTTCTGTCATCAGAAACTCAAACGAAGACAACAACGAA
AACAAGAACAACAAGAGACACAGTGACAATAACAGAGACAATGACT
ACAATGATGATGACCAAAAGAATCACCAAAACTCTGGATCCCACAAC
AACAACAACAACAACAACAACAATGACAACGGAAACAACAACTACA
ACAACAACGCCCGTGCATGGAAACAAGGACAAGTCTATGAATACCA
AATCCAAGGCCGCACCCTAGCTGCTCTTCATGATGTAGCTGACCAATA
CACCGGAACCATCATCAAAGCCACCCTCAAGGTTCAACCCAGAAATC
AAGACTCAGTCTTGGCTTGGGTCACCAACGCTAGACACTCTGATGTTC
ACGCCAACCTAACCAATGGCTGGAACCAAGAGATCCCAGACAAATAC

CTCAACTACCAAAACTGGCAACTCAGTGACAAACCATTCGCCATCCA
ATTCAAGAATGGAGTTGTTAAGAATCTGCAAGTCAGCCAAAACCTGC
CAACCTGGGAACTGAACATCATCAAGAGTATTGTCAGTCAACTCCAA
GTTGACACCAGAGCCGAAAATGAAGTCAGCTCGCGTCTCAACCAGAA
ACCCAAGAACGGCAAACCTTTCGGAACCTTCAAGACCATGGAAGACA
CCGTCACTGGAGAATGTGAAACCTTGTACGACATCAAACCTCTGCAAC
AAGTTGAACAACAAAACAAACCTCAACTCGCCCCCATGCCAAACCTC
AAGGGATCAAACGGAGACCTTATTGACATCATCAAGACCAAGAACTT
CAGTCGTTGTGACTCCAGAATCGAATACCACTTCGGACTTCCCGGAAG
CAACGATGTTGAACCATCAAGCAACCAAGTTCTTAACTTCTTGTCCAG
ATCTTCAACCAGCAGAGTCATCATTGCTGGAGACCTGAGCCACTACAC
CATCCAATCCTCAGTCACCACCGACAAAATTGTCATCAGTCCCGAACT
CTACAACAAACAAAAGGGAATGGTTGTCAGTCGCCTGAACGTTACTC
TGTCCAACGTCCACTCTGCTGCTCAAAACAATGCCCCAGCTCTCCCAT
CCAACGTCAACAAAGTTGATGATTTGATCTACGAATACAACCCAGCTT
CCCCTGACAACGAATCTGCCCAAAACAACAACGGCAAGAACAACCA
CCACAACGACGATGACTCATCCTCATCTTCTTCTTCAAGTTCTTCCAGC
TCCTCTTCTTCATCCTCCAGCAGTTCTGACAGTTCCTCCTCTTCTTCCTC
GTCCTCCTCCTCAGACAGCTCATCATCTTCTGACAGTTCCAGCTCTTCC
TCTGACAGCTCAAACTCTTCCTCCAACAGTTCCGAAAGTGACGAGAGA
CAAAACAACAAGAGACGCAATAACAACAGCGAAGAAAACAACAAG
AAGAACCACAACAACAGAAGCAACGAAAGTAGAAACAACAAAAAC
CACAATGGCAACAGCAATGAAAACAGAAACAAGAACAACGAAGAC
AACGACTACACCATGAGCTCCGAACAAGGAAAGAACAGAAGAATCA
GATCCCGTCGTTCTGTCATCAGAAACTCAAACGAAGACAACAACGAA
AACAAGAACAACAAGAGACACAGTGACAATAACAGAGACAATGACT
ACAATGATGATGACCAAAAGAATCACCAAAACTCTGGATCCCACAAC
AACAACAACCATCACAACTCTGGATCTAATGACAACAAGAACAACCA
TATGTCCAACAAAAACTGGAAACAAGGTCGCGATTCTTCCAGCTCCA
GCTCTAGCTCTTCTGACTCCAGCAGCTCTTCATCCAGCTCCTCAGAAA
GTGGAAGTTCTTCTTCCTCTTCATCTTCTTCCTCCAGCAGCAGCGAAGA
ATTCCACCAACCTGAACCCAAGCTTAACCAAGCCCCCAAATCTCCTTT
CATGCCCTACTTCATCGGAAACAAAGGAAACTCCATCGAATCTTCCAA
ACAAATCGACGGAGTTGCTGTCACCAAATCTTTGGCCCAACAAATTGG
TCAAGAAATCCAAGAACCCAACACCTTGGCTGAACACAAAACCCTTT
CCAAATTCACCATCCTCGCCGGAGTTATCCGCACCATGAACGCCAAAC
AACTGGAAGCCGCCACTCACGAACTCTACTACCAACAAAACAAGGCC
TCTTCATCCAGCCAATCCGATGCCACCAAATTAACCGCATGGAAAGCT
TACCGTGACGCCGTTGCTCAAGCAGGAACTGGTCCCGCTTTGATGGCC
ATGAAACAATGGATTGAATCCGGCAAAGTCGAAGGAGAAGAAGCTG
CTGAATTATTGGCTGTTCTCCCCAACACCGCCAGATACCCAACCCGTG
AATACATCAAGGAATTCTTCAACCTTGCCACCAGCTCTCAAGTCACCA
AACAAGCTCACCTCAACACCTCTGCTATCCTCTCCGTCGCCAGTTTGG

CAAGAAAAGCCCAAGTTGACTCTGACAACTCACACAACCAATACCCA
GTTCATGCTTTCGGACCCCTGTCATCCAAAAACAGCAAAGACATCACT
GAAAGATACATCCCATACTTGGCCAACAAACTCAAGGAAGCTACCAG
AAACCAAGACAGTCTGAAAGCTCAAGTCTACATCAAAGCTCTCGGAA
ACTTAGGACATACCGCCGTCCTCGCTGTCTTCAAACCATACCTTGAAG
GAAAAGCCCCAGCCACCAACTTCCAACGTCTTTCAATGGTTGCCGCCA
TGGACCAAGTTGCCAGATTATCACCAAAATCTGTCCAACCTGCTCTCT
TCAACATCTACCTCAACACCGGAGAAAGCCACGAATTACGTTGTGCTG
CTGTATTCCAACTCATGAAGACCTACCCATCCGCTCAATTGCTCCAAC
GTATGGCTGCATTCACTGAACAAGACATGAGCAAACAAGTTAACTCT
GCTGTCAAATCCGCCATCGAAAGCGCTGCTGAACAACAACACCCCAA
ACTTCAAGAATTGTAA

>RNA-XM_026832896.1, gene id: LOC103523199, amino acid sequence

MSSEQGKNRRIRSRRSVIRNSNEDNNENKNNKRHSDNNRDNDYNDDDQ
KNHQNSGSHNNNNNNNNNNNNNNDNGNNNYNNNARAWKQGQVYEY
QIQGRTLAALHDVADQYTGTIIKATLKVQPRNQDSVLAWVTNARHSDVH
ANLTNGWNQEIPDKYLNYQNWQLSDKPFAIQFKNGVVKNLQVSQNLPT
WELNIIKSIVSQLQVDTRAENEVSSRLNQKPKNGKPFGTFKTMEDTVTGEC
ETLYDIKPLQQVEQQNKPQLAPMPNLKGSNGDLIDIIKTKNFSRCDSRIEYH
FGLPGSNDVEPSSNQVLNFLSRSSTSRVIIAGDLSHYTIQSSVTTDKIVISPELY
NKQKGMVVSRLNVTLSNVHSAAQNNAPALPSNVNKVDDLIYEYNPASPD
NESAQNNNGKNNHHNDDDSSSSSSSSSSSSSSSSSSSDSSSSSSSSSSDSSSSS
DSSSSSSDSSNSSSNSSESDERQNNKRRNNNSEENNKKNHNNRSNESRNN
KNHNGNSNENRNKNNEDNDYTMSSEQGKNRRIRSRRSVIRNSNEDNNE
NKNNKRHSDNNRDNDYNDDDQKNHQNSGSHNNNNNNNNNNDNGNN
NYNNNARAWKQGQVYEYQIQGRTLAALHDVADQYTGTIIKATLKVQPR
NQDSVLAWVTNARHSDVHANLTNGWNQEIPDKYLNYQNWQLSDKPFAI
QFKNGVVKNLQVSQNLPTWELNIIKSIVSQLQVDTRAENEVSSRLNQKPK
NGKPFGTFKTMEDTVTGECETLYDIKPLQQVEQQNKPQLAPMPNLKGSN
GDLIDIIKTKNFSRCDSRIEYHFGLPGSNDVEPSSNQVLNFLSRSSTSRVIIAG
DLSHYTIQSSVTTDKIVISPELYNKQKGMVVSRLNVTLSNVHSAAQNNAPA
LPSNVNKVDDLIYEYNPASPDNESAQNNNGKNNHHNDDDSSSSSSSSSSS
SSSSSSSDSSSSSSSSSSSDSSSSSDSSSSSSDSSNSSSNSSESDERQNNKRRNNN
SEENNKKNHNNRSNESRNNKNHNGNSNENRNKNNEDNDYTMSSEQGK
NRRIRSRRSVIRNSNEDNNENKNNKRHSDNNRDNDYNDDDQKNHQNSG
SHNNNNHHNSGSNDNKNNHMSNKNWKQGRDSSSSSSSSDSSSSSSSSSES
GSSSSSSSSSSSSSEEFHQPEPKLNQAPKSPFMPYFIGNKGNSIESSKQIDGVA
VTKSLAQQIGQEIQEPNTLAEHKTLSKFTILAGVIRTMNAKQLEAATHELY
YQQNKASSSSQSDATKLTAWKAYRDAVAQAGTGPALMAMKQWIESGKV
EGEEAAELLAVLPNTARYPTREYIKEFFNLATSSQVTKQAHLNTSAILSVAS
LARKAQVDSDNSHNQYPVHAFGPLSSKNSKDITERYIPYLANKLKEATRN
QDSLKAQVYIKALGNLGHTAVLAVFKPYLEGKAPATNFQRLSMVAAMDQ

VARLSPKSVQPALFNIYLNTGESHELRCAAVFQLMKTYPSAQLLQRMAAF
TEQDMSKQVNSAVKSAIESAAEQQHPKLQEL

LOC113469177 *Vitellogenin-like* [*Diaphorina citri* (Asian citrus psyllid)]

Gene ID: 113469177, updated on 10-Nov-2018, 1002 bp *Vg5*

>RNA-XM_026826680.1, gene id: LOC113469177, gene sequence

ATTTCAGCATGGAAACAAGGACAAGTCTATGAATACCAAATCCAAGG
CCGCACCCTAGCTGCTCTTCATGATGTAGCTGACCAATACACCGGAAC
CATCATCAAAGCCACCCTCAAGGTTCAACCCAGAAATCAAGACTCAG
TCTTGGCTTGGGTCACCAACGCTAGACACTCTGATGTTCACGCCAACC
TAACCAATGGCTGGAACCAAGAGATCCCAGACAAATACCTCAACTAC
CAAAACTGGCAACTCAGTGACAAACCATTCGCCATCCAATTCAAGAA
TGGAGTTGTTAAGAATCTGCAAGTCAGCCAAAACCTGCCAACCTGGG
AACTGAACATCATCAAGAGTATTGTCAGTCAACTCCAAGTTGACACCA
GAGCCGAAAATGAAGTCAGCTCGCGTCTCAACCAGAAACCCAAGAA
CGGCAAACCTTTCGGAACCTTCAAGACCATGGAAGACACCGTCACTG
GAGAATGTGAAACCTTGTACGACATCAAACCTCTGCAACAAGTTGAA
CAACAAAACAAACCTCAACTCGCCCCCATGCCAAACCTCAAGGGATC
AAACGGAGACCTTATTGACATCATCAAGACCAAGAACTTCAGTCGTT
GTGACTCCAGAATCGAATACCACTTCGGACTTCCCGGAAGCAACGAT
GTTGAACCATCAAGCAACCAAGTTCTTAACTTCTTGTCCAGATCTTCA
ACCAGCAGAGTCATCATTGCTGGAGACCTGAGCCACTACACCATCCA
ATCCTCAGTCACCACCGACAAAATTGTCATCAGTCCCGAACTCTACAA
CAAACAAAAGGGAATGGTTGTCAGTCGCCTGAACGTTACTCTGTCCAA
CGTCCACTCTGCTGCTCAAAACAATGCCCCAGCTCTCCCATCCAACGT
CAACAAAGTTGATGATTTGATCTACGAATACAACCCAGCTTCCCCTGA
CAACGAATCTGCCCAAAACAACAACGGCAAGAACAACCACCACAAC
GACGATGAC
>RNA-XM_026826680.1, gene id: LOC113469177, amino acid sequence

ISAWKQGQVYEYQIQGRTLAALHDVADQYTGTIIKATLKVQPRNQDSVLA
WVTNARHSDVHANLTNGWNQEIPDKYLNYQNWQLSDKPFAIQFKNGVV
KNLQVSQNLPTWELNIIKSIVSQLQVDTRAENEVSSRLNQKPKNGKPFGTF
KTMEDTVTGECETLYDIKPLQQVEQQNKPQLAPMPNLKGSNGDLIDIIKTK
NFSRCDSRIEYHFGLPGSNDVEPSSNQVLNFLSRSSTSRVIIAGDLSHYTIQSS
VTTDKIVISPELYNKQKGMVVSRLNVTLSNVHSAAQNNAPALPSNVNKV
DDLIYEYNPASPDNESAQNNNGKNNHHNDDD

LOC103524089 *Vitellogenin receptor* [*Diaphorina citri* (Asian citrus psyllid)]

Gene ID: 103524089, updated on 15-Jul-20195950 bp *VgR*

>RNA-XM_026833263.1, gene id: LOC103524089, gene sequence

ATGGGTCCTTTGTATTTAGCAACGTGCTTCCTGTTTCTGCTTTACCCACC
TTATCAACATGTGGTACGAGGTGAAGATGTAACGTGTTTGGATAGTCA
GTTCCTGTGTCACAACTCTACAGTGTGTATCGAGAAGTCTCAAGTGTG
TGATGGAGTTATACAGTGTCAATACGAAGACGATGAGTTTGATTGTGC
TTTCTTCATGATTGCAGATATCTACCTGTGTAAGTCGCCTAACTATTAC
AAATGTGAAGACGGCTCGTGTATAACATCCTCCTTCTTGTGTGATGGA
AGTGACGACTGTCTGGATGGTTCTGATGAAGCTAACTGTACGAGTAGT
AGTGCTCTCTCCAGTCAGCAAGAGTGTGATAGTATGGAATTCAAATGC
AGCAATGGCAAGTGTATAACACAACATTGGGTGTGTGATGGGGAAGA
TGATTGTGGTGACGGGAGTGATGAAACTGAGATGGCGTGTAAGAAGG
TAGGTGACATCCTGTATATGAAGATTGATGGAGTTATACAGTGTCAAT
ACGAAGACGATGAGTTTGATTGTGATATCTACCTGTGTAAGTCGCCTA
ACTATTACAAGTGTGAAGACGGCTCGTGTATAACATCCTCCTTCTTGTG
TGATGGAAGTGACGACTGTCTGGATGGTTCTGATGAAGCTAACTGTAC
GAGTAGTAGTGCTCTCTCCAGTCAGCAAGAGTGTGATAGTATGGAATT
CAAATGCAGCAATGGCAAGTGTATAACACAACATTGGGTGTGTGATG
GGGAAGATGATTGTGGTGACGGGAGTGATGAAACTGAGATGGCGTGT
AAGAAGAACACGGATTGTATTGGGGACTTCCTATGTCGGAATCATAA
CTGTATAGTTAAGGATTGGGTGTGTGATGGTGTCGATGATTGTCAGGA
TAATTCAGATGAAGAAAATTGTCCACACAAAGATGAGTTCCCCATGTC
GGAATGCACGTCAGAGCATCAGAGGTTCAAATGTCACGATGAAAGTC
ATTGTGTTGAGTTTCATAAGTTATGTGATAATCATACTGACTGTATTGA
TGGATCTGATGAAGGGGGACAGTGCTCTACTGTGTGTACGTATTCATG
TAGGAAAAAATACTATCAACCTAGTTTCAAATGGAGCAACAAGTACA
GCTTTGATGGATTCAAGAGTTTTCCCGCGAGCTTTAAGATAGAAGATT
GGAATGAGATGGAATTCATGCGGGTGACAGCGGCTAAAGGTTACGCT
CACATGGGACCTAACCCTTGTGCAGACTTTGGAGGGTGCAGTGATATT
TGTCTATTGGCAGAAATAGTCGTGTCGGCAGGTCTAGGTTCCCCGGAA
GACTTGTCTGTAGACTGGCTCACACACAATATTTACTTCACGGATCTTA
AGGCGCAACATATTGGAGTGTGCAATAATAAGGGAGAGCATTGTGTG
ATCATAGTCAACGCAGATATAGATCGACCACGGGGTATAACGCTGCT
CCCTGTAGAACGCATAATGTTCTGGTCAGACTGGGGTAAAGTTCCCAT
GATAGCTACCAGCGGCATGGATGGCTCTAACCCGCGACCTTTCATTAG
CGACAGTATCCACTGGCCTAATGATGTCACTGTAGACTACTTCGGCTC
TAGACTGTACTGGATAGATGCTAAGCTGAAGATTATTGAAACTGTCAA
GCTAGATGGAACTGACAGAAGGACTGTTCTCTCGGATCGTGTCAAGC
ACCCATTTTCCATCGCCATCTTCGAAGACCAGATCTACTGGAGTGATT

GGGTTTCCATGGAGATATTGGCGTGTAACAAATTCACCGGTAGAGGGC
GACATGTCATGCGGAAAGAGAGAGATGATCAGATATATGGACTGCAT
GTTTACCATCCCGTCATGACTAATGTTCCTAATACTAGGAAAAGTACA
ATTTTAAAACTGACCATCATCATCAGTGATATCTCAGCTAAGAAGATC
TCCTATCTGAATTTAGAGACCAGTGAGGTATCTCTCATACTACGAGGT
TCAGTGGGTACTATCACTGCTATGGATTACGATAACCTGGGCTCTAAC
CTGTACTGGATTGATACGGAACGAGGTACTGTGGAGGTGTTGAATATG
AAGAGACTTGTGCGTACCGTGCTACTACAGAACCTTACCGAGCTGCCT
ATCGCCCTTGCACTTGTGCCTAAGGAAGGATTCATGTTCGTGGCATTCT
CACACAAGCATCACATCCACATTGATCGTATTCGTATGGATGGCTCAC
TACAACAACGTACACATGTAATAGAGGATGGTTTAGTGGGACCTCAC
ATTGTAATGCACTATGATGAAGATTTAGAGCGAGTGTTCTGGGCAGAT
GCGTTCACTGGAGCTATAGAGAGTACTGATAGTCAAGGTCTGGACCGT
CTCTCATACACCAATGTATCATCTCCCATGGGCTTGGCTACTGTTAGCA
ACGATCTATTCTGGACCAGCTATCAACCTAGTTTCAAATGGAGCAACA
AGTACAGCTTTGATGGATTCAAGAGTTTTCCCGCGAGCTTTAAGATAG
AAGATTGGAATGAGATGGAATTCATGCGGGTGACAGCGGCTAAAGGT
TACGCTCACATGGGACCTAACCCTTGTGCAGACTTTGGAGGGTGCAGT
GATATTTGTCTATTGGCAGGCAAAACACATGTATGCGCATGTCCTGCT
GGTAAAATCCTCAATGCTAATGGTTTAACATGTGAAGATCTGCCCAAG
TGTTCCAATGAGAAACAGTTCCAATGTCATAATGGTCAATGTATAGCA
CTTCATCTGGTTTGCAACGGGCACAATGATTGTGTCGGTGGAGAAGAC
GAGCAAGCATCGTGCACGCCCTCAGCACATCTGAATTGTTCTGAACTT
CACTTCCCCTGTATGAACGGGGAGCGATGTATTGATCTAACGCTCAGA
TGTAACAATGAGTTTGATTGTGAGGATAAATCTGATGAGTTCCATTGT
AATAACACGGTCAAGACGTGCTCTGGAGAGTTTGATTTCCAGTGTGAT
TCAGGCGAATGTATTGGACGTCATTTCCTATGTGATAAATCTAACGAC
TGTATGGATGGCTCAGATGAGAACCCTAAGCATTGTGTGAACTGTTCT
AGTAGTGAGTTTAGATGCGCTACAGGATCTTGTATACCTGCTAGCTGG
ATTTGTGATGGAGCCCCGGATTGTACAGACAATTCAGATGAGATGTAT
TGTGATAAGAAAGAAGTTTGTGGAACCTCAGCATTTACCTGTAGCAAT
GAGAATTGTGTTCCTCTCAAGCTGAAATGTGATGGCAATGATGACTGT
GGTGATAGATCCGATGAACAAGATTGTCCTAGCATAGATCTTACTGGG
CAGTGTCTTCCACCCAACTTCCTCTGTTCTACACTACCCAGCCTATGTC
TACCTGCCAATGCCAAGTATGAGATGTCATGTGGTTCATGTGATACTC
ACTCTGAGTTTGAATGTCCTGAGTCACACCAATGCATACCCAACTCCT
GGCTATGTGACCATCAACCTGATTGTACTGGGGGAGAAGATGAGAAC
CCAGCACTCTGTACTCAGAGGTACACTGCACCACGTCTATCATCTACT
ACACCCAGGTTTAGAGATGTGTTGCCATGTTCCGAGTTTTCCTGTGAGA
ATGGTCAGTGTCTAAGATATTCACAAGTGTGTGATAAACACCCTGACT
GTATGGATGGTACAGATGAAGGTGGACGTTGCAACACTGGATGCTCT
ACCATAGATTGTGACGTGTTTTATGAATTCTCGGTATGCGCATGTCCTG
CTGGTAAAATCCTCAATGCTAATGGTTTAACATGTGAAGATCTGCCCA

AGTGTTCCAATGAGAAACAGTTCCAATGTCATAATGGTCAATGTATAG
CACTTCATCTGGTTTGCAACGGGCACAATGATTGTGTCGGTGGAGAAG
ACGAGCAAGCATCGTGCACGCCCTCAGCACATCTGAATTGTTCTGAAC
TTCACTTCCCCTGTATGAACGGGGAGCGATGTATTGATCTAACGCTCA
GATGTAACAATGAGTTTGATTGTGAGGATAAATCTGATGAGTTCCATT
GTAATAACACGGTCAAGACGTGCTCTGGAGAGTTTGATTTCCAGTGTG
ATTCAGGCGAATGCATTGGACGTCATTTCCTATGTGATAAATCTAACG
ACTGTATGGATGGACCTCCTATGGAAGTTCTATACACAATGAATGATC
AGCTTCGCAAAGTATCTTCCTCTCATCTCAAGATCATGTTTGAATATCC
TGGAGTTCAGGTTAAAGGTCTTGACATTGATATTAGAAAAGGGCTGGT
CTATTGGTCGTCTGCAGAGTCAGGCATGGTCACTCAGTTCAACATGAA
AACTCTATCACGTCGCCTCTACATTTCCGGCCTTAGTCGTCCTGAGCGG
CTCGCATTAGATTGGATTCGTAACCTAGTGTATATTGTAGAGTCAGAG
AGGAAGATCATGGCGTGTCACATGGAACGACATGTGTGCGTTAGGGT
GTACTCTTCACTGGATAATATTCATATCTCTGCCCTCTCTGTAGATCCT
ATCAATGGGTATTTATTCTGGGCGGAAACCTCCTGGCTGATGTGGGAC
GCACCAGTAGGTGTCATCAAGCGTAGTGACCTCTCCGGTAGTAATGTG
ACGACAATAGTAGAAGGTAGCGTATCCCATGTGACCTCAATCGCTATT
AATGACATCAAGCGACATCTGTACTGGACAGACTCGGCTAAGAAACT
AATAGAACAGACTCAAATGGATGGAGCTTATAGGAAGAGGATACTAG
ATACCAAGGTCCCCGCACTTCAGTTGCACCTGTTCGAGGATATCCTAT
ATTACGTGACGCCCATCATACAGGGTGCGCCATTCATCAAGTGCCACC
TCTATGGGAATCTACAGGGGTCTTGTGAGCAGCTGGATATACATGTGG
CCAACCCTGTCACTCATTTCACCATATCACAATTGTCTAGACAGAGAA
TGGGTCCTAACAAGTGTACCAACTTCTCCTGTAGTCATATGTGTTTAGA
AAGTTCTACAGGGCCCGTGTGTATATGTCCTGATGGTAGTAAAGTCAC
CGGTAATCAGTACTGTGGAGTTAATATAGAGGATCCGATGTTAAGGA
ACCCTGCCATATTCCAGAACCATACCCAGGTGGAGGGAGAGGATATG
TCATCAGGTTCTGGTGTGGGTGTGCTGTTTATTATTGGTCTCATTCTAGT
GGTGCTCTCCATATCTGCAGTCTATCATTTGTGTCTCAGGAAACGAGTG
GCAAACCTAGTACCCAAGATTCACTTCAGGAATCCCGTATTCAATGGA
GGATACTCAGAGAGTAACTTAAGGAATGTTCCTATGAGTCAGAACTTC
TATTCTCCTACCTCTCCTACTATGGTCTTACCTAATATTTATGAAAATGT
AGTAGAGCCAGTCCCTCCTGTTCAGATCAAGTATGCGAATGAGATGA
GTATCAAACATGGAGAAGAAGATTTCTGGAATACTAGGAGACATTCA
GAGTCCTCTACTGGTACTGATTATGCTGAGATACAAGACAACCCCAAA
CTACCTCTCCTCTAG

>RNA-XM_026833263.1, gene id: LOC103524089, amino acid sequence

MGPLYLATCFLFLLYPPYQHVVRGEDVTCLDSQFLCHNSTVCIEKSQVCDG
VIQCQYEDDEFDCAFFMIADIYLCKSPNYYKCEDGSCITSSFLCDGSDDCLD
GSDEANCTSSSALSSQQECDSMEFKCSNGKCITQHWVCDGEDDCGDGSDE
TEMACKKVGDILYMKIDGVIQCQYEDDEFDCDIYLCKSPNYYKCEDGSCIT

SSFLCDGSDDCLDGSDEANCTSSSALSSQQECDSMEFKCSNGKCITQHWVC
DGEDDCGDGSDETEMACKKNTDCIGDFLCRNHNCIVKDWVCDGVDDCQ
DNSDEENCPHKDEFPMSECTSEHQRFKCHDESHCVEFHKLCDNHTDCID
GSDEGGQCSTVCTYSCRKKYYQPSFKWSNKYSFDGFKSFPASFKIEDWNEM
EFMRVTAAKGYAHMGPNPCADFGGCSDICLLAEIVVSAGLGSPEDLSVDW
LTHNIYFTDLKAQHIGVCNNKGEHCVIIVNADIDRPRGITLLPVERIMFWS
DWGKVPMIATSGMDGSNPRPFISDSIHWPNDVTVDYFGSRLYWIDAKLKII
ETVKLDGTDRRTVLSDRVKHPFSIAIFEDQIYWSDWVSMEILACNKFTGRG
RHVMRKERDDQIYGLHVYHPVMTNVPNTRKSTILKLTIIISDISAKKISYLNL
ETSEVSLILRGSVGTITAMDYDNLGSNLYWIDTERGTVEVLNMKRLVRTVL
LQNLTELPIALALVPKEGFMFVAFSHKHHIHIDRIRMDGSLQQRTHVIEDG
LVGPHIVMHYDEDLERVFWADAFTGAIESTDSQGLDRLSYTNVSSPMGLA
TVSNDLFWTSYQPSFKWSNKYSFDGFKSFPASFKIEDWNEMEFMRVTAAK
GYAHMGPNPCADFGGCSDICLLAGKTHVCACPAGKILNANGLTCEDLPK
CSNEKQFQCHNGQCIALHLVCNGHNDCVGGEDEQASCTPSAHLNCSEL
HFPCMNGERCIDLTLRCNNEFDCEDKSDEFHCNNTVKTCSGEFDFQCDSG
ECIGRHFLCDKSNDCMDGSDENPKHCVNCSSSEFRCATGSCIPASWICDGA
PDCTDNSDEMYCDKKEVCGTSAFTCSNENCVPLKLKCDGNDDCGDRSDE
QDCPSIDLTGQCLPPNFLCSTLPSLCLPANAKYEMSCGSCDTHSEFECPESH
QCIPNSWLCDHQPDCTGGEDENPALCTQRYTAPRLSSTTPRFRDVLPCSEF
SCENGQCLRYSQVCDKHPDCMDGTDEGGRCNTGCSTIDCDVFYEFSVCA
CPAGKILNANGLTCEDLPKCSNEKQFQCHNGQCIALHLVCNGHNDCVG
GEDEQASCTPSAHLNCSELHFPCMNGERCIDLTLRCNNEFDCEDKSDEFH
CNNTVKTCSGEFDFQCDSGECIGRHFLCDKSNDCMDGPPMEVLYTMNDQ
LRKVSSSHLKIMFEYPGVQVKGLDIDIRKGLVYWSSAESGMVTQFNMKTLS
RRLYISGLSRPERLALDWIRNLVYIVESERKIMACHMERHVCVRVYSSLDNI
HISALSVDPINGYLFWAETSWLMWDAPVGVIKRSDLSGSNVTTIVEGSVSH
VTSIAINDIKRHLYWTDSAKKLIEQTQMDGAYRKRILDTKVPALQLHLFEDI
LYYVTPIIQGAPFIKCHLYGNLQGSCEQLDIHVANPVTHFTISQLSRQRMGP
NKCTNFSCSHMCLESSTGPVCICPDGSKVTGNQYCGVNIEDPMLRNPAIFQ
NHTQVEGEDMSSGSGVGVLFIIGLILVVLSISAVYHLCLRKRVANLVPKIHF
RNPVFNGGYSESNLRNVPMSQNFYSPTSPTMVLPNIYENVVEPVPPVQIKY
ANEMSIKHGEEDFWNTRRHSESSTGTDYAEIQDNPKLPLL

3. Base sequence of the dsRNA synthesis gene fragment

The original template of *Vg4* gene for synthesizing *dsVg4* (*Vitellogenin-A1-like*, *Diaphorina citri Vg4*, 477bp).

ATGGCCATGAAACAATGGATTGAATCCGGCAAAGTCGAAGGAGAAG
AAGCTGCTGAATTATTGGCTGTTCTCCCCAACACCGCCAGATACCCAA

CCCGTGAATACATCAAGGAATTCTTCAACCTTGCCACCAGCTCTCAAG
TCACCAAACAAGCTCACCTCAACACCTCTGCTATCCTCTCCGTCGCCA
GTTTGGCAAGAAAAGCCCAAGTTGACTCTGACAACTCACACAACCAA
TACCCAGTTCATGCTTTCGGACCCCTGTCATCCAAAAACAGCAAAGAC
ATCACTGAAAGATACATCCCATACTTGGCCAACAAACTCAAGGAAGC
TACCAGAAACCAAGACAGTCTGAAAGCTCAAGTCTACATCAAAGCTC
TCGGAAACTTAGGACATACCGCCGTCCTCGCTGTCTTCAAACCATACC
TTGAAGGAAAAGCCCCAGCCACCAACTTCCAACGTCTTTCAATGGTTG
CC

The original template of *VgR* gene for synthesizing *dsVgR* (*Vitellogenin receptor, Diaphorina citri VgR*, 346bp).

TGTTCGATGACGATTGTCCTAGCATAGATCTTACTGGGCAGTGTCTTCC
ACCCAACTTCCTCTGTTCTACACTACCCAGCCTATGTCTACCTGCCAAT
GCCAAGTATGAGATGTCATGTGGTTCATGTGATACTCACTCTGAGTTTG
AATGTCCTGAGTCACACCAATGCATACCCAACTCCTGGCTATGTGACC
ATCAACCTGATTGTACTGGGGGAGAAGATGAGAACCCAGCACTCTGT
ACTCAGAGGTACACTGCACCACGTCTATCATCTACTACACCCAGGTTT
AGAGATGTGTTGCCATGTTCCGAGTTTTCCCAA

4. Gene qRT-PCR amplification, melting curves and standard curve

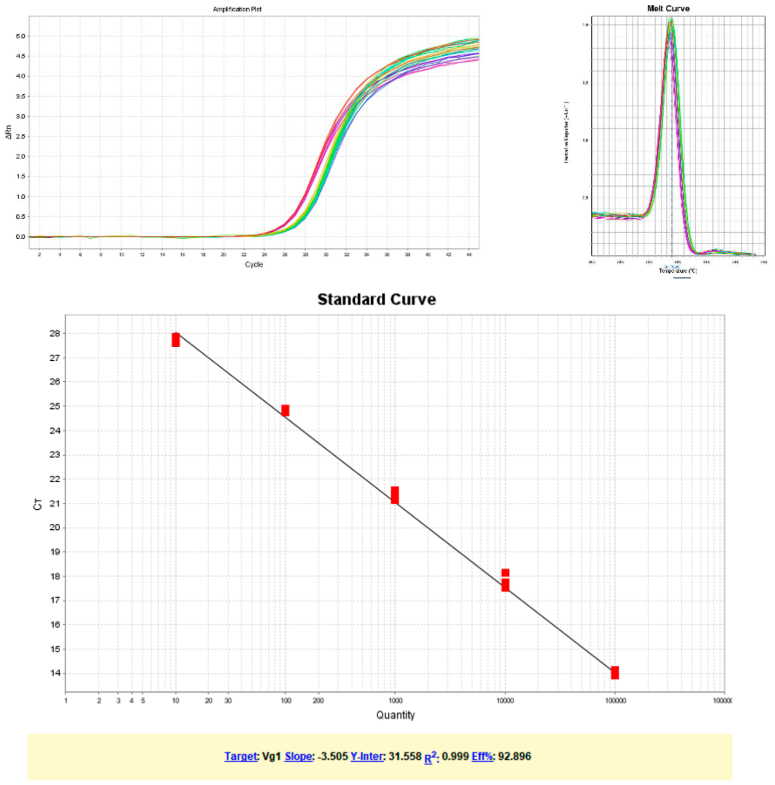

Figure S1. *Vg1* gene qRT-PCR amplification, melting curve and standard curve.

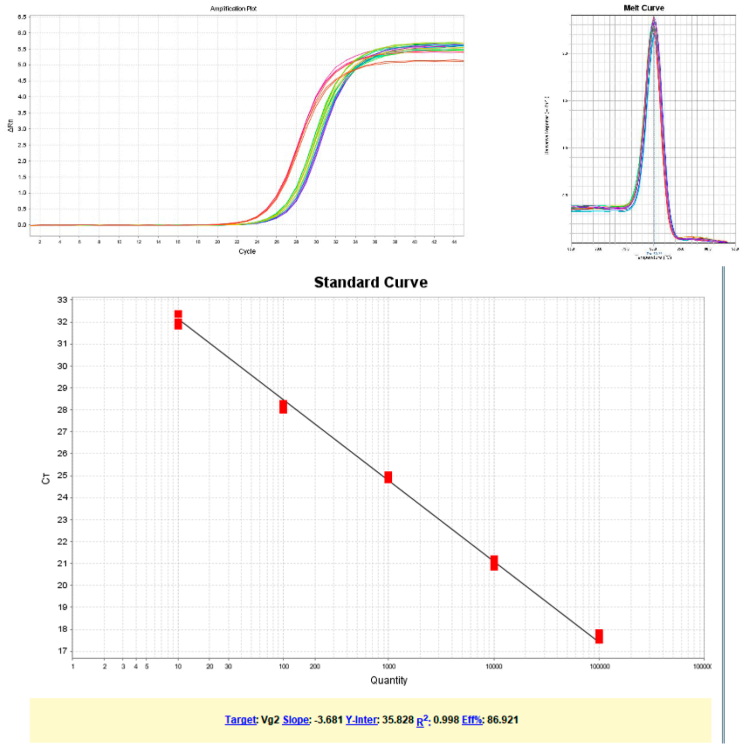

Figure S2. *Vg2* gene qRT-PCR amplification, melting curve and standard curve.

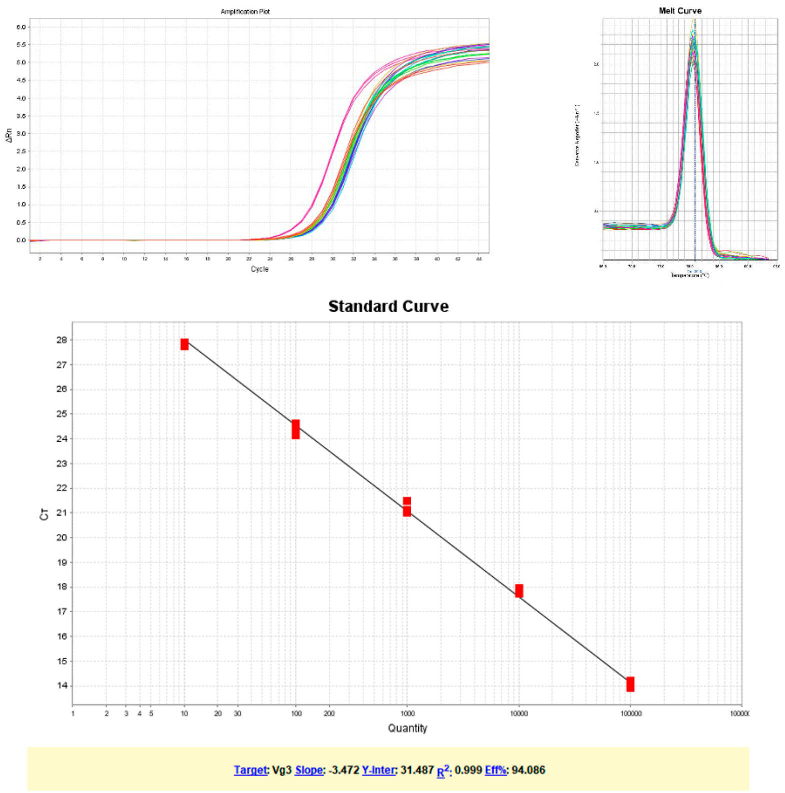

Figure S3. *Vg3* gene qRT-PCR amplification, melting curve and standard curve.

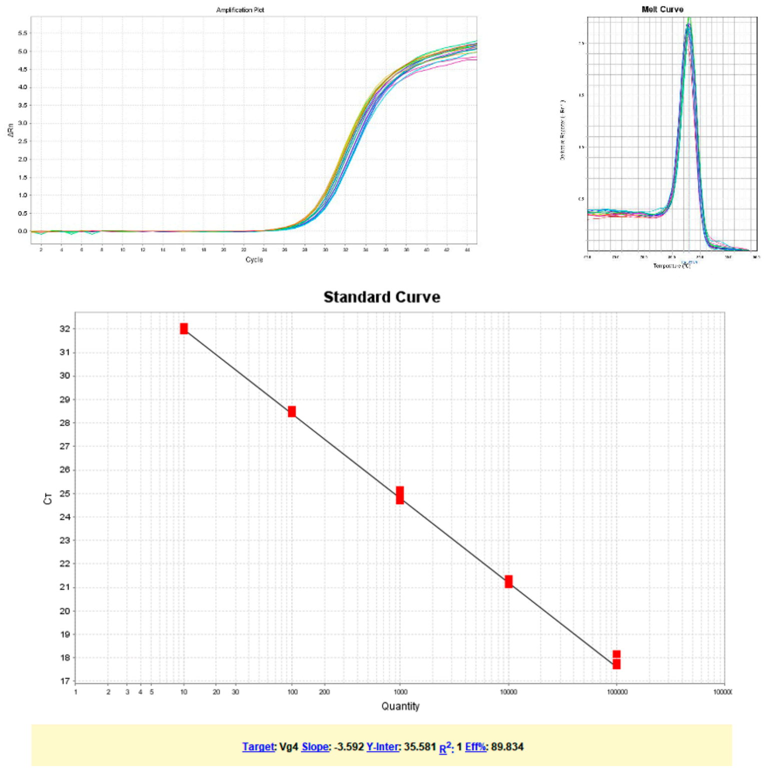

Figure S4. *Vg4* gene qRT-PCR amplification, melting curve and standard curve.

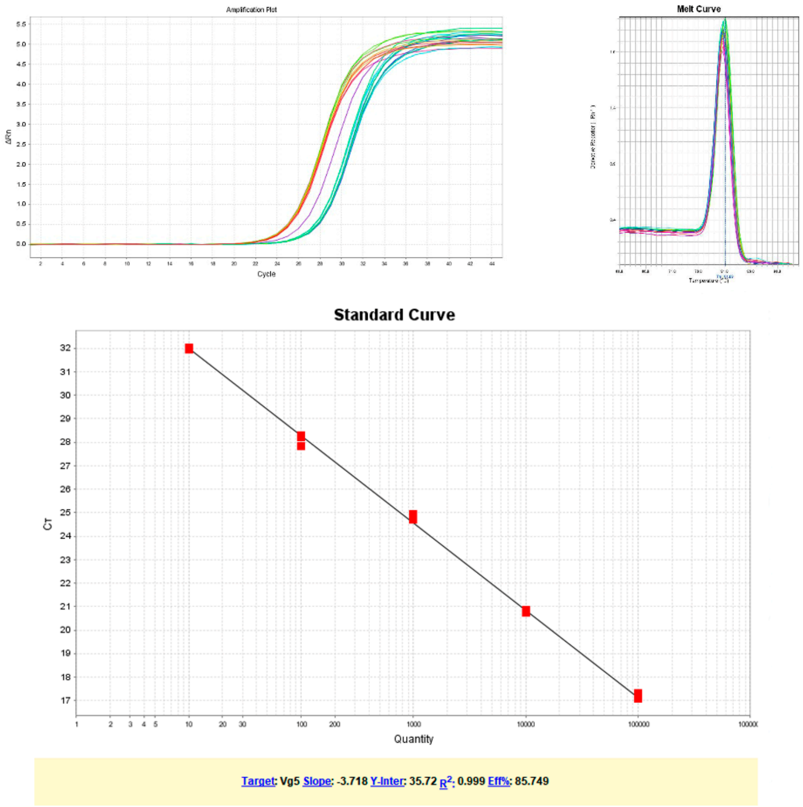

Figure S5. *Vg5* gene qRT-PCR amplification, melting curve and standard curve.

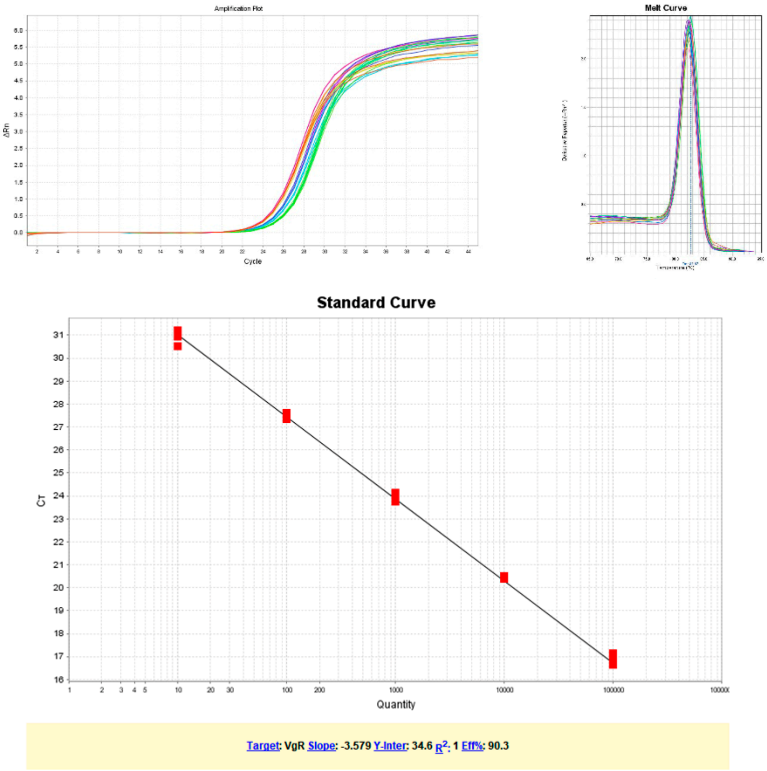

Figure S6. *VgR* gene qRT-PCR amplification, melting curve and standard curve.

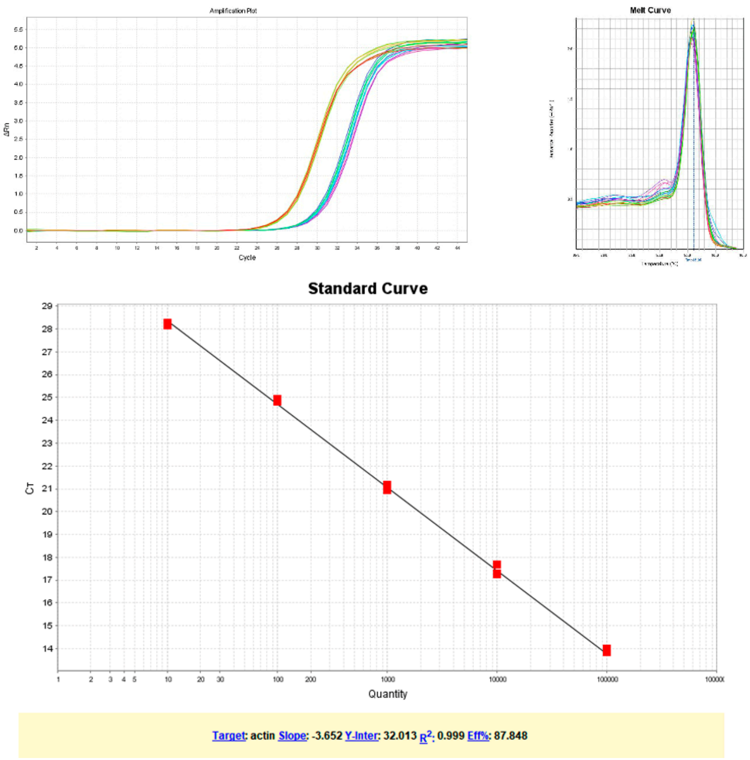

Figure S7. *Actin* gene qRT-PCR amplification, melting curve and standard curve.

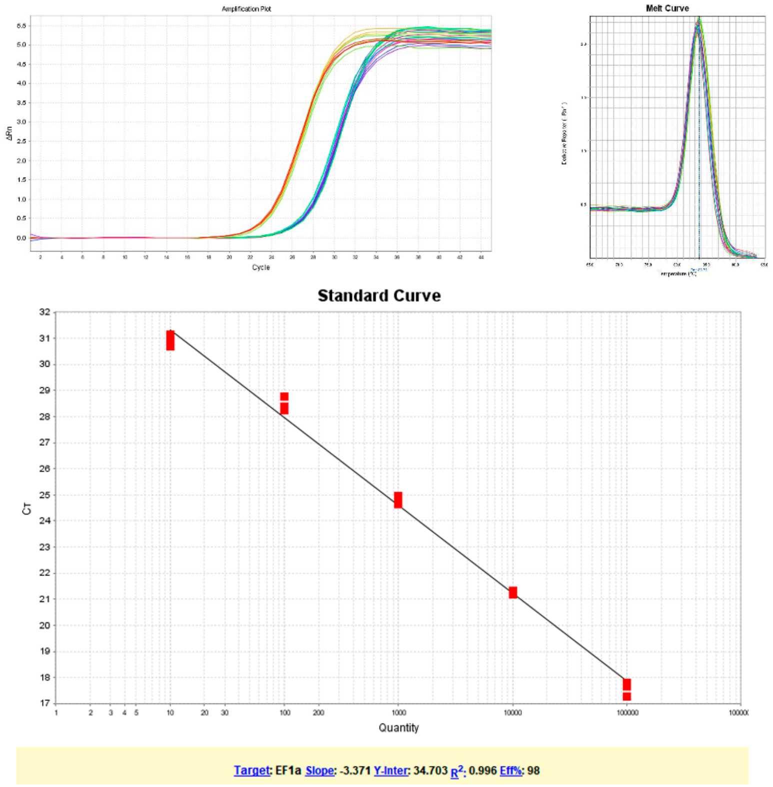

Figure S8. *EF1a* gene qRT-PCR amplification, melting curve and standard curve.

5. Gene qRT-PCR CT value

Table S1. Gene qRT-PCR CT value.

Day	*Vg1* CT	*Vg2* CT	*Vg3* CT	*Vg4* CT	*Vg5 CT*	*VgR CT*	*Actin CT*	*EF1a CT*
0d	24.94873428	22.75183678	26.47142982	25.19622803	27.06477737	20.59365082	26.9736042	23.33963013
0d	25.01749802	22.85720253	26.50704765	25.29025841	27.21294403	20.68389702	26.8793087	23.42202377
0d	25.16228294	22.96621704	26.31925964	25.17372513	27.19555283	20.90293694	27.46232414	23.46230698
0d	25.12845612	22.85710907	26.60997009	24.81414795	27.08278656	20.77813339	27.08530426	23.53865242
0d	24.96143341	22.50120926	25.95926094	25.08118629	26.89682198	20.44390106	26.98298073	23.57659912
0d	24.89261055	22.66380119	26.15975189	25.09945297	27.15281105	20.57632637	26.88469887	23.44706345
0d	25.21280861	22.69161224	26.34324265	25.03744125	27.09087753	20.69828796	27.01093674	23.80102921
0d	24.93103218	22.56198883	26.08125496	25.00600433	27.0983429	20.69668198	27.23193741	23.58708382
0d	24.84268761	22.72075081	26.6985569	25.05811119	27.00277901	20.55097771	26.84642982	23.39916039
5d	27.93235779	26.43834114	29.7549572	27.84651566	30.16800499	24.66574478	30.60059357	27.16849327
5d	27.87569046	26.16842079	29.65537643	27.5286274	29.69154739	24.44473267	29.86686516	27.17089272
5d	27.96655846	26.04594421	29.40833473	27.79067612	29.64967346	24.44216919	29.91456795	26.65607643
5d	27.91558456	25.998806	29.67028809	27.84092903	29.45690727	24.32195091	29.6682682	27.1748085
5d	28.0123539	26.15637016	29.53677559	27.55717468	29.50609589	24.46832657	30.04549217	27.78093529
5d	27.93632126	25.96339607	29.64740181	27.52476883	29.50733185	24.51693344	30.11965752	26.73011398
5d	28.02326775	26.12858009	30.01619339	27.77684975	29.54183578	24.26713562	29.84647942	26.86956024
5d	27.92865944	26.14599037	29.69327927	27.90342903	29.76215935	24.3178215	29.71775246	27.10988808
5d	28.07893372	26.08295631	29.7409687	27.84447098	29.61937141	24.36387062	29.68535805	27.04083633
10d	26.68354607	24.58478737	28.54885292	26.57701874	28.42265892	22.6593647	30.16335487	27.03154373
10d	26.6619606	24.54385567	28.3010807	26.37608719	28.74909592	22.61968231	30.41903114	26.99606133
10d	26.91464996	24.43784904	28.19001007	26.49063873	28.156147	22.57152557	30.93958473	26.9867897
10d	26.80030632	24.62303925	28.49336433	26.39945221	28.31510162	22.78244781	30.90787315	27.01813126
10d	26.71784782	24.54260254	28.49042702	26.44403648	28.30152321	22.92193985	30.56395531	27.06668663
10d	26.88014412	24.61717796	28.71618843	26.55246735	28.27522087	22.70014763	30.26793289	27.09067917
10d	26.6649971	24.66030884	28.3116951	26.83336639	28.31499672	22.69623756	30.2225132	27.01032829
10d	26.73066139	24.69795609	28.59296036	26.62352371	28.16214371	22.79756546	30.76542854	26.83530045
10d	26.73542595	24.55860138	28.70732689	26.68116188	28.11654282	22.82234192	30.52171326	26.84765434
15d	27.70413589	26.72437859	28.51879692	26.65166473	28.45782471	25.98581696	29.4341507	25.39731598
15d	27.55155182	26.90730476	28.5560112	26.75084686	28.63685036	26.10466766	29.38488197	25.33146477
15d	27.53401756	26.64383888	28.54452515	26.68101883	28.75173759	26.23982048	29.3368969	25.29359055
15d	27.5267849	26.71636772	28.79047585	26.41587448	29.44042397	26.08816338	29.22824478	25.59041595
15d	27.78935051	26.52742767	28.66528702	26.52425575	28.35902023	25.8547802	29.76744652	25.27636147
15d	28.0245266	26.91308975	28.87747955	26.44569969	28.586092	25.99301338	29.55724525	25.12909126
15d	27.76030159	26.49595642	28.54719162	26.91699791	28.49294281	25.9522686	29.98989296	25.2114296
15d	27.83808517	26.67699051	29.15690422	26.6183567	28.47975731	26.01529312	29.09719276	25.01746368
15d	27.96569824	26.70627785	28.87790871	26.67712402	28.6246357	25.95808411	29.44139671	25.06327248
20d	28.06305695	27.0293541	29.6335659	27.26136589	29.08550453	25.47206116	30.9817276	27.5173893
20d	28.03521919	27.02003479	29.49723816	26.99034309	29.16581345	25.26180458	30.66134644	27.51173782
20d	28.24602127	27.21001244	30.01580238	27.44259644	29.03890991	25.57123375	31.41500282	27.22365761
20d	28.26141548	27.09266663	28.97202682	26.97196198	28.77052307	25.11715889	30.71622467	27.45004654
20d	28.33005905	27.23184395	29.34995651	27.19591141	28.82785797	25.25090599	30.54999542	27.35832214
20d	28.01738739	27.18985176	29.48192596	27.08253288	28.76827049	25.66820145	31.07453156	27.197258
20d	28.31327248	27.33383942	29.28801727	27.36400032	28.92158508	25.21438789	30.49965668	27.7547245
20d	27.95388603	27.1000309	29.06111526	26.96614265	28.94690132	25.24401283	30.54255676	27.44871902
20d	28.31393433	27.11393929	29.52449226	27.06271172	28.87032318	25.29954147	31.15953064	27.31273079
25d	25.97773933	25.07642746	27.0715847	25.08891869	26.95294952	24.45975494	28.79800224	30.22975159

Table S1. Gene qRT-PCR CT value (Continued).

Day	*Vg1* CT	*Vg2* CT	*Vg3* CT	*Vg4* CT	*Vg5 CT*	*VgR CT*	*Actin CT*	*EF1a CT*
25d	25.7849330	24.9715404	26.97228432	24.968853	26.85069466	24.4975853	29.1511344	29.87985229
25d	25.9749679	24.9856948	27.22876358	25.21003532	26.92006874	24.42596245	28.97010231	30.07688713
25d	25.86518097	24.99221039	27.23902321	25.25810623	27.35229683	24.61724472	29.59800911	30.04125786
25d	25.7707901	25.16409492	27.05282021	25.18174553	26.85745239	24.61261559	29.39775085	30.12535667
25d	25.80096054	25.1203289	27.09632492	24.89844704	26.9620266	24.7169342	29.06714439	30.3989296
25d	25.75681305	25.00934601	26.87998772	25.00748825	26.93049812	24.66915321	28.93668938	30.09051323
25d	25.70521927	25.0483799	26.71223831	25.01706505	27.00268555	24.7820797	28.74168777	30.14302254
25d	25.62021828	25.12672997	26.49957275	24.92163849	27.14938164	24.64432716	29.23291016	30.42858696
30d	25.35346222	22.98750877	26.22921371	24.87933922	26.74993896	21.45557976	28.56038857	24.98677254
30d	25.50875854	22.87644958	25.98228264	24.56023598	26.63790131	21.4847393	28.52107239	25.16342545
30d	25.28662682	22.98882294	25.98841476	24.63946724	26.58243561	21.33288574	28.41692543	25.30775642
30d	25.30166435	22.67841721	26.74900246	24.578722	26.71564293	21.3916893	28.51629448	24.93468094
30d	25.49851418	22.69921684	26.10073662	24.60544968	26.45503998	21.28834343	28.46153641	25.00835228
30d	25.4424839	23.08349037	25.91170502	24.7218895	26.61409187	21.37858963	28.18375778	25.12962723
30d	25.52972984	22.87388039	26.78709221	24.61678314	26.49873161	21.42393684	28.1726799	24.81313133
30d	25.5414772	22.88592529	25.83154869	24.83813095	26.5731678	21.51166534	28.67874146	25.03516579
30d	25.40067101	22.95261955	25.92791939	24.76352882	26.62865257	21.49782753	28.37917328	25.23766518

Table S2. Gene RNAi qRT-PCR CT value.

Day	*Vg4* CT	*VgR CT*	*Actin CT*	*EF1a CT*
5d	19.963	29.479	22.240	25.410
5d	19.987	29.081	22.373	25.684
5d	19.953	29.234	22.416	25.443
5d	20.020	29.081	22.491	25.347
5d	19.936	29.167	22.312	25.431
5d	19.876	29.132	22.882	25.470
5d	19.954	29.321	22.289	25.311
5d	19.869	29.169	22.310	25.290
5d	19.806	28.960	22.249	25.383
10d	19.963	28.986	22.240	23.766
10d	19.987	29.075	22.373	23.891
10d	19.953	28.336	22.416	23.868
10d	20.020	29.043	22.491	23.829
10d	19.936	28.987	22.312	23.844
10d	19.876	29.138	22.882	23.826
10d	19.954	28.662	22.289	23.870
10d	19.869	27.911	22.310	23.861
10d	19.806	28.674	22.249	23.950
15d	30.780	27.966	25.710	24.636
15d	30.941	27.857	25.708	24.925
15d	34.893	27.869	25.707	24.691
15d	31.311	27.868	25.857	24.781
15d	31.265	27.770	25.889	24.723
15d	30.972	27.612	25.566	24.751
15d	30.814	27.612	25.685	24.709
15d	31.363	27.785	25.894	24.713
15d	30.968	27.752	25.603	24.739
20d	26.989	28.882	27.286	25.631
20d	27.155	29.282	27.765	25.737

Table S2. Gene RNAi qRT-PCR CT value (Continued).

Day	*Vg4* CT	*VgR CT*	*Actin CT*	*EF1a CT*
20d	27.050	29.318	27.822	25.780
20d	27.508	29.146	27.417	25.558
20d	27.172	28.977	27.637	25.589
20d	26.966	27.887	27.396	25.660
20d	27.041	29.134	27.496	25.511
20d	27.202	29.143	27.258	25.450
20d	27.097	28.900	27.590	25.608
25d	28.293	30.003	25.717	24.962
25d	28.607	29.674	25.611	24.949
25d	28.730	29.691	26.236	24.884
25d	28.574	29.929	25.643	24.953
25d	28.739	30.004	26.172	25.217
25d	28.574	28.888	25.593	25.058
25d	28.169	29.948	25.393	24.940
25d	28.252	29.636	25.607	24.906
25d	28.308	29.962	25.479	24.988
30d	31.444	29.138	27.954	25.510
30d	31.093	29.185	28.006	25.939
30d	30.918	29.293	28.051	25.848
30d	31.048	29.238	27.972	25.937
30d	30.954	29.257	28.068	25.752
30d	30.988	29.311	27.950	26.177
30d	30.955	29.008	28.055	26.108
30d	31.282	29.405	28.025	25.941
30d	30.503	29.352	28.201	26.055

5. Different development stages ovarian phenotype characteristics

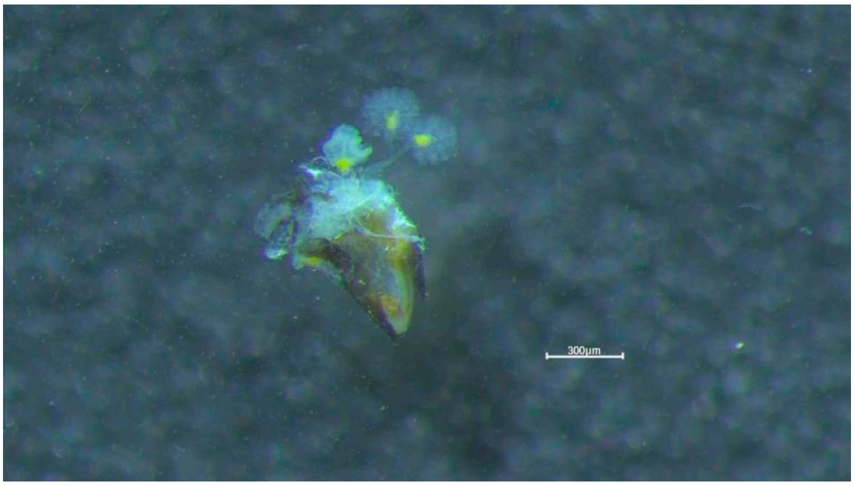

Figure S9. Ovaries at the newly eclosion stage (0d).

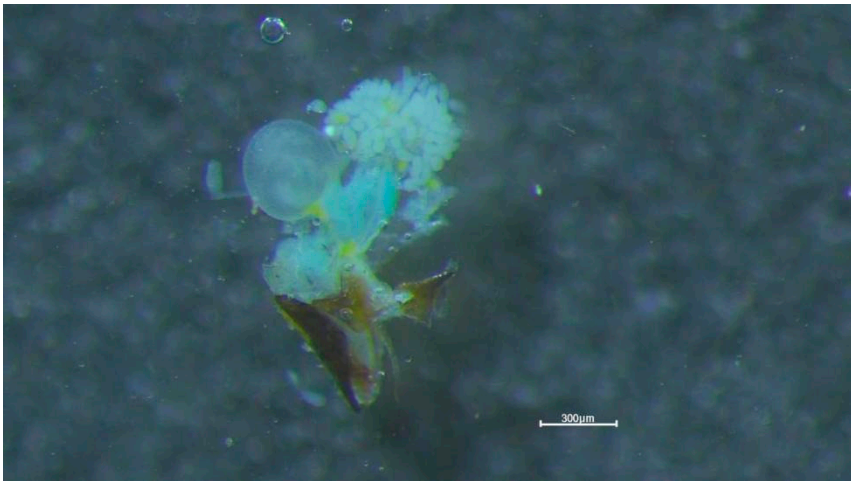

Figure S10. Ovaries at the 5-day ovarian development stage.

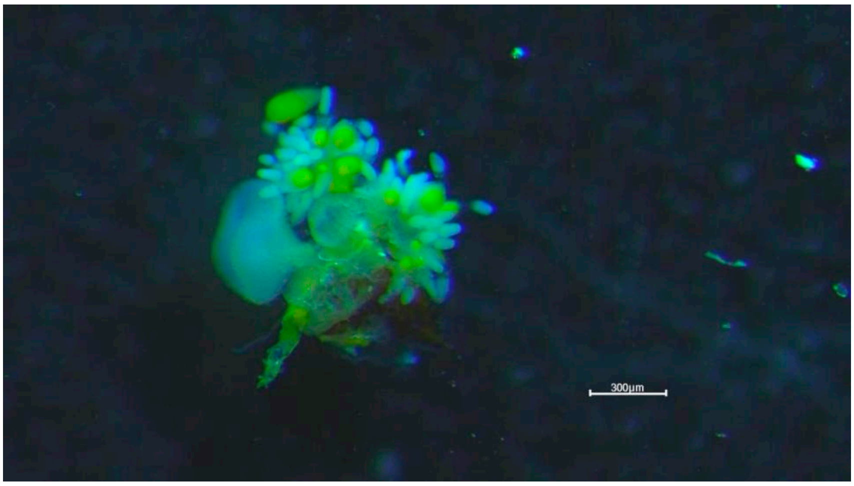

Figure S11. Ovaries at the 10-day ovarian development stage.

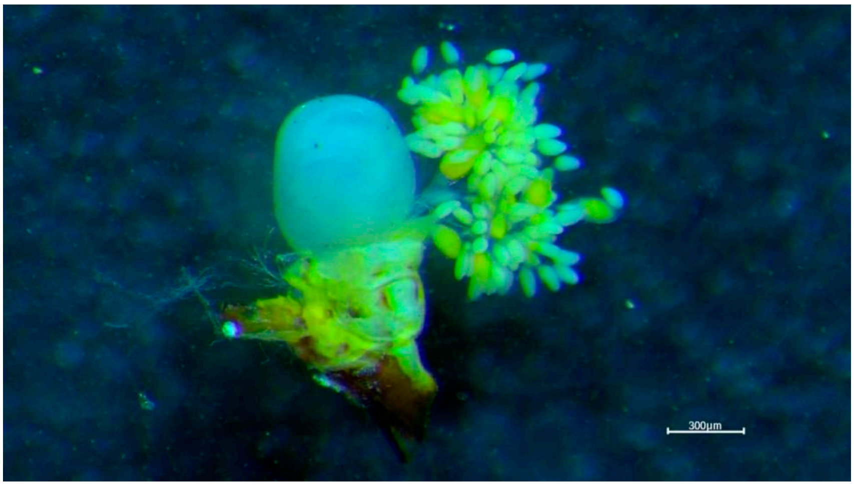

Figure S12. Ovaries at the 15-day ovarian development stage.

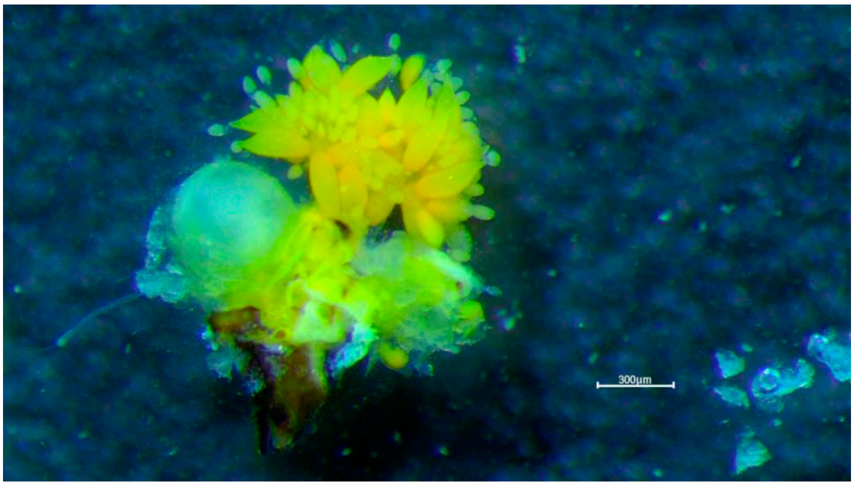

Figure S13. Ovaries at the 20-day ovarian development stage.

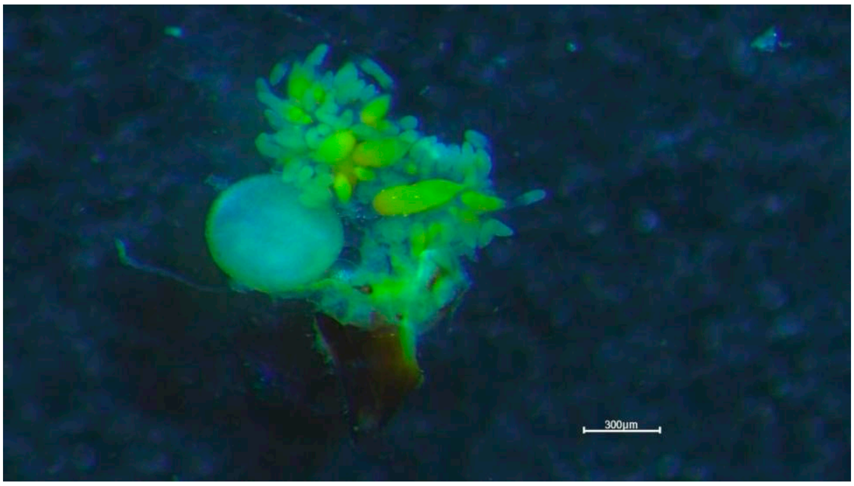

Figure S14. Ovaries at the 25-day ovarian development stage.

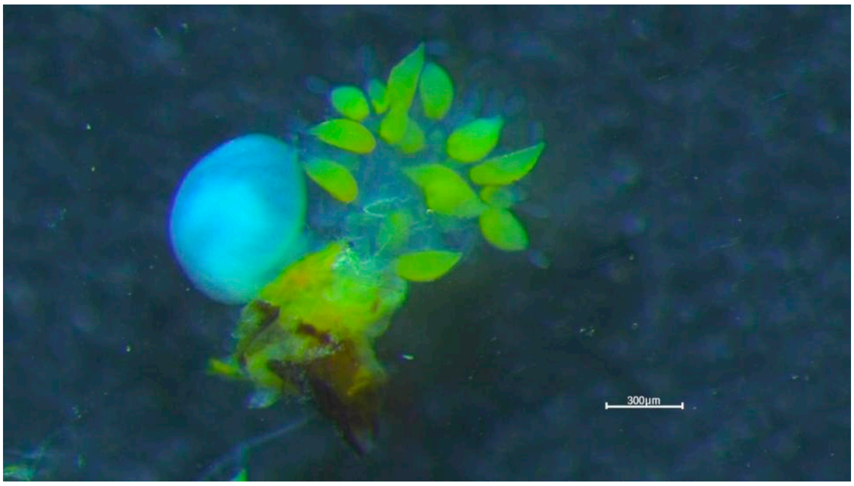

Figure S15. Ovaries at the 30-day ovarian development stage.

6. Stability of dsRNA in *M. odorifera* Shoots

Gel electrophoresis was employed to assess the stability of ds*Vg4*, ds*VgR*, and ds*GFP* in treated *M. odorifera* shoots over six days. Clear gel electrophoresis bands were observed at 1–6 days in the *M. odorifera* shoots treated with ds*GFP*, indicating the feasibility of IPS for delivering dsRNA (Figure S16). Ds*Vg4* and ds*VgR* displayed clear bands at 1–2 days, suggesting that ds*Vg4* and ds*VgR* maintained a relatively complete double-stranded structure during this period. Once *D. citri* females fed on the *M. odorifera* shoots for 1–2 days, ds*Vg4* and ds*VgR* readily entered the female body. The bands became blurred after 3–6 days, signifying that ds*Vg4* and ds*VgR* started to decompose at this stage. However, traces of bands in ds*Vg4* gel electrophoresis indicated that the structures of some ds*Vg4* and ds*VgR* remained intact at 3–6 days (Figure S16). Chromas analysis of gene sequencing results for ds*Vg4* and ds*VgR* was consistent with the template gene used to synthesize dsRNA (Figure S17).

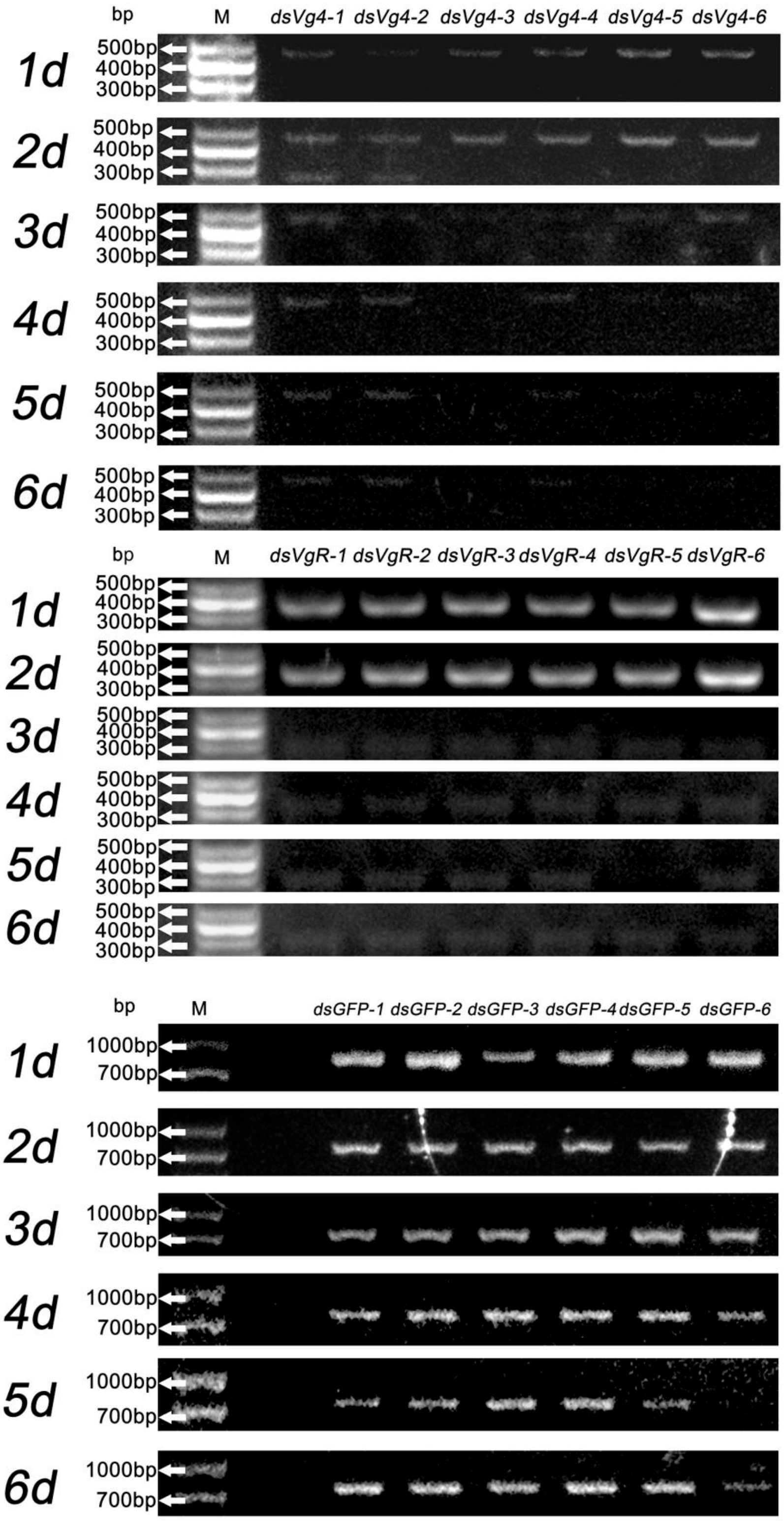

Figure S16. *DsVg4*, *dsVgR*, and *dsGFP* were absorbed by tender *M. odorifera* shoots for six days, and gel electrophoresis was used to detect the persistence of *dsVg4*, *dsVgR*, and *dsGFP* after each day. The gel electrophoresis bands of *dsVg4* were relatively clear at 1–6 d; the gel electrophoresis bands of *dsVgR* were relatively clear at 1–2 d, but blurry at 3–6 d; the gel electrophoresis bands of *dsGFP* were relatively clear at 1–6 d. The *dsVg4*-1, 2, 3, 4, 5, 6/*dsVgR*-1, 2, 3, 4, 5, 6/*dsGFP*-1, 2, 3, 4, 5, 6 represent the six replicates for each treatment.

Vg4 sequencing base sequence, the sequencing result was basically consistent with the base sequence of *Vg4* gene.

AATGGCAGTCGAGGAGAGAGCTGCTGATTATTGGCTGTTCTCCCCAAC
ACCGCCAGATACCCAACCCGTGAATACATCAAGGAATTCTTCAACCTT
GCCACCAGCTCTCAAGTCACCAAACAAGCTCACCTCAACACCTCTGCT
ATCCTCTCCGTCGCCAGTTTGGCAAGAAAAGCCCAAGTTGACTCTGAC
AACTCACACAACCAATACCCAGTTCATGCTTTCGGACCCCTGTCATCC
AAAAACAGCAAAGACATCACTGAAAGATACATCCCATACTTGGCCAA
CAAACTCAAGGAAGCTACCAGAAACCAAGACAGTCTGAAAGCTCAA
GTCTACATCAAAGCTCTCGGAAACTTAGGACATACCGCCGTCCTCGCT
GTCTTCAAACCATACCTTGAAGGAAAAGCCCCAGCCACCAACTTCCA
ACGTCTTTCATGGGTTGCCCAAAACA

The original template of *Vg4* gene for synthesizing *dsVg4* (*Vitellogenin-A1-like, Diaphorina citri Vg4*, 477bp).

ATGGCCATGAAACAATGGATTGAATCCGGCAAAGTCGAAGGAGAAGA
AGCTGCTGAATTATTGGCTGTTCTCCCCAACACCGCCAGATACCCAACC
CGTGAATACATCAAGGAATTCTTCAACCTTGCCACCAGCTCTCAAGTC
ACCAAACAAGCTCACCTCAACACCTCTGCTATCCTCTCCGTCGCCAGT
TTGGCAAGAAAAGCCCAAGTTGACTCTGACAACTCACACAACCAATA
CCCAGTTCATGCTTTCGGACCCCTGTCATCCAAAAACAGCAAAGACAT
CACTGAAAGATACATCCCATACTTGGCCAACAAACTCAAGGAAGCTAC
CAGAAACCAAGACAGTCTGAAAGCTCAAGTCTACATCAAAGCTCTCG
GAAACTTAGGACATACCGCCGTCCTCGCTGTCTTCAAACCATACCTTGA
AGGAAAAGCCCCAGCCACCAACTTCCAACGTCTTTCAATGGTTGCC

VgR sequencing base sequence, the sequencing result was basically consistent with the base sequence of *VgR* gene.

GATGGCAATGATGACTGTGGTGATAGATCCGATGAACAAGATTGTCCT
AGCATAGATCTTACTGGGCAGTGTCTTCCACCCAACTTCCTCTGTTCTA
CACTACCCAGCCTATGTCTACCTGCCAATGCCAAGTATGAGATGTCATG
TGGTTCATGTGATACTCACTCTGAGTTTGAATGTCCTGAGTCACACCAA
TGCATACCCAACTCCTGGCTATGTGACCATCAACCTGATTGTACTGGGG
GAGAAGATGAGAACCCAGCACTCTGTACTCAGAGGTACACTGCACCA
CGTCTATCATCTACTACACCCAGGTTTAGAGATGTGTTGCCATGTTCCG

AGTTTT

The original template of *VgR* gene for synthesizing *dsVgR* (*Vitellogenin receptor*, *Diaphorina citri VgR*, 346bp).

TGTTCGATGACGATTGTCCTAGCATAGATCTTACTGGGCAGTGTCTTCC
ACCCAACTTCCTCTGTTCTACACTACCCAGCCTATGTCTACCTGCCAAT
GCCAAGTATGAGATGTCATGTGGTTCATGTGATACTCACTCTGAGTTTG
AATGTCCTGAGTCACACCAATGCATACCCAACTCCTGGCTATGTGACC
ATCAACCTGATTGTACTGGGGGAGAAGATGAGAACCCAGCACTCTGTA
CTCAGAGGTACACTGCACCACGTCTATCATCTACTACACCCAGGTTTAG
AGATGTGTTGCCATGTTCCGAGTTTTCCCAA

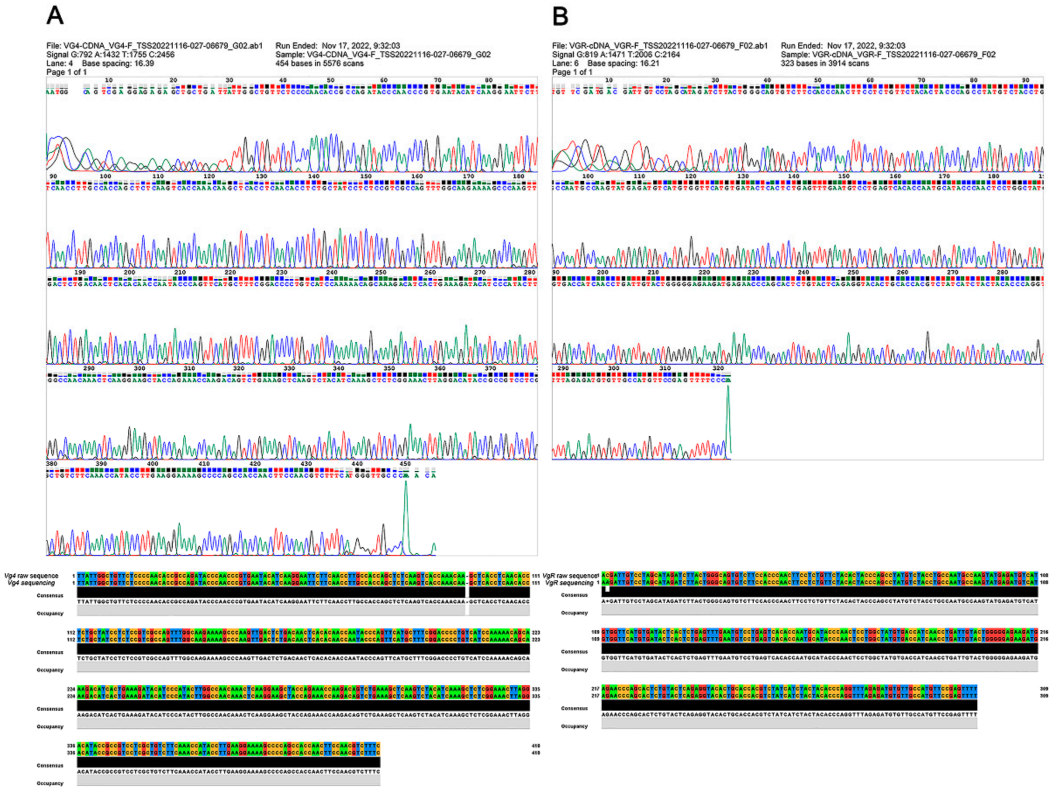

Figure S17. After *M. odorifera* shoots total RNA of absorbing *dsVg4* and *dsVgR* were extracted, the sequencing results of *Vg4* and *VgR* gene interference fragments were cloned using cDNA as template. (A) Base sequence and base feasibility of *Vg4* gene, sequence alignment between the *Vg4* sequencing sequence and the original sequence. (B) Base sequence and base feasibility of *VgR* gene, sequence alignment between the *VgR* sequencing sequence and the original sequence.
